# Chartarlactams U-X: Novel Phenylspirodrimanes from a Marine Derived Fungus *Stachybotrys* sp. SZU-W23 with Anti-Inflammatory Activity Mediated by the NF-κB/ROS Signaling Pathways

**DOI:** 10.3390/md23050216

**Published:** 2025-05-20

**Authors:** Yanhua Wu, Lanyi Lu, Peng Zhang, Liyan Wang

**Affiliations:** 1 Shenzhen Key Laboratory of Microbial Genetic Engineering, College of Life Sciences and Oceanography, Shenzhen University, Shenzhen 518060, China; wuyanhua@szu.edu.cn (Y.W.); 2200251008@email.szu.edu.cn (L.L.); 2 College of Physics and Optoelectronic Engineering, Shenzhen University, Shenzhen 518060, China; 3 State Key Laboratory of Discovery and Utilization of Functional Components in Traditional Chinese Medicine, Natural Products Research Center of Guizhou Province, Guiyang 550014, China

**Keywords:** *Stachybotrys* sp., phenylspirodrimanes, anti-inflammation, NF-κB pathway

## Abstract

In this investigation, the anti-inflammatory potential of phenylspirodrimanes (PSDs) produced by the marine-derived fungal strain *Stachybotrys* sp. SZU-W23 was systematically explored. A total of 15 PSDs were successfully isolated. Among them, four novel compounds, designated as chartarlactams U-X, were precisely characterized using NMR, HRESIMS, and ECD analyses. Specifically, compound **10** exhibited the most potent inhibitory effect on nitric oxide production in LPS-stimulated RAW 264.7 macrophages within the 0.3–30 μM concentration range, with an IC_50_ value of 12.4 μM. Additionally, MTT assays revealed no detectable cytotoxicity at these concentrations. Mechanistic studies revealed that compound **10** effectively suppressed ROS generation, likely inactivating the NF-κB signaling pathway and consequently downregulating pro-inflammatory mediators, including iNOS, IL-6, and IL-1β.

## 1. Introduction

Triisopentenyl phenolic compounds, a class of natural products featuring benzene rings and triisopentenyl moieties, exhibit broad pharmacological activities against inflammation, thrombosis, and oxidative stress [1,2,3,4,5]. Notably, their anti-inflammatory properties hold therapeutic promise for treating inflammation-associated pathologies [6,7].

**Figure 1:**
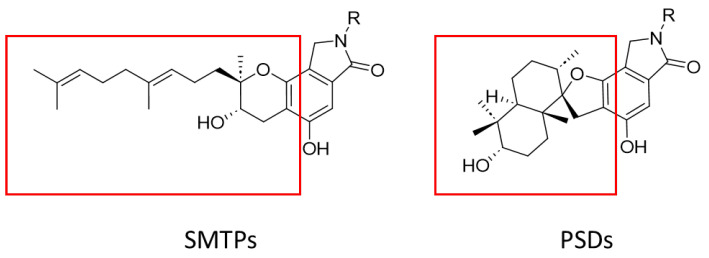
Two primary structural types of triisopentenyl phenolic compounds.

Triisopentenyl phenolic compounds can be classified into two main structural classes: *Stachybotrys microspora* triprenyl phenols (SMTPs) and phenylspirodrimanes (PSDs) (Figure 1). These two classes both belong to the triisopentenyl phenolic compound category, sharing the common feature of containing benzene rings and triisopentenyl moieties. However, they exhibit distinct structural differences. SMTPs feature a benzopyran core structure, which is composed of a γ-lactam moiety, an isoprenoid side chain, and an N-substituent of the γ-lactam group [8]. In contrast, PSDs are characterized by a phenylpropylfuran skeleton, with two six–membered rings formed by isopentenyl side chains [4,9].

In terms of bioactivity, SMTPs demonstrate thrombolytic, anti-inflammatory, and antioxidant properties [2,8]. Notably, SMTP-7, a representative of this class, has advanced to phase II clinical trials, demonstrating effective thrombolysis with minimal adverse effects [10,11]. In contrast, PSDs exhibited a broader spectrum of pharmacological activities. They have been reported to possess anti-HIV activity, antihyperlipidemic effects, and inhibitory actions against viral proteases [9,12]. These findings underscore the substantial research and developmental potential of PSDs, which extends beyond the well-characterized SMTPs.

PSDs are predominantly biosynthesized by fungi of the genera *Stachybotrys* spp. and *Memnoniella* spp., resulting in the production of structurally diverse homologs such as stachybotrysins [13], stachybotrylactones [3,10,14], stachybotrins [15], and stachybotrylactams [6].

In our laboratory, we have been continuously engaged in researching anti-inflammatory compounds derived from marine fungi [16,17,18,19,20,21]. During the preliminary screening of anti-inflammatory substances, we found that the fermentation extract of the marine-derived fungal strain *Stachybotrys* sp. SZU-W23 exhibited remarkable inhibitory activity against nitric oxide (NO) production. Consequently, an in-depth investigation was conducted on the anti-inflammatory secondary metabolites produced by this strain. In this study, fifteen PSDs analogs, including four novel chartarlactam analogs (**1**–**4**), were isolated and characterized from a marine derived *Stachybotrys* sp. SZU-W23. Structural elucidations of the novel compounds were achieved through high-resolution mass spectrometry (HR-MS), nuclear magnetic resonance (NMR) spectroscopy (^1^H, ^13^C, 2D), and electronic circular dichroism (ECD) comparisons. Known compounds were identified through a comparative analysis of their properties with those reported in the published literature [6,9,22]. Additionally, anti-inflammatory and antioxidant activities were tested for these compounds.

## 2. Results

### 2.1. Identification of PSDs

**Figure 2:**
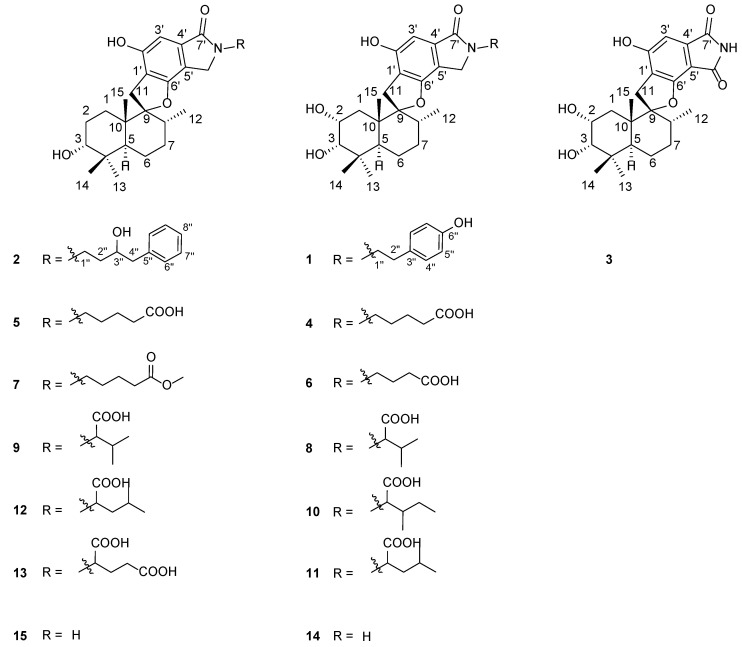
Structures of compounds **1**–**15**.

Compounds **1**–**4** were all isolated as white powders. The molecular formula of **1** was determined as C_31_H_39_NO_6_ by HR-ESI-MS, which requires 13 degrees of unsaturation. The ^1^H and ^13^C NMR spectra of **1** (Table 1 and Figures S1–S8) are similar to those reported for chartarlactams P, indicating the presence of the same C-2 hydroxylated phenylspirodrimane skeleton [9]. The planar structure of compound **1** was confirmed through a detailed analysis of 2D NMR spectra (Figure 1). The remaining NMR data revealed the presence of two methylene signals and a 4-hydroxy-phenyl moiety (Table 1). An ethyl linkage was identified through the ^1^H-^1^H COSY correlation of H-1″ and H-2″, and the N-linked 4-hydroxyphenylethyl side chain was established according to the HMBC correlations, including those from H-1″ (*δ*_H_ 3.61) to C-7′ (*δ*_C_ 167.7) and C-8′ (*δ*_C_ 47.4), from H-2″ (*δ*_H_ 2.79) to C-4″ (*δ*_C_ 129.9), and from H-4″ (*δ*_H_ 7.04) to C-2″ (*δ*_C_ 33.6) and C-3″ (*δ*_C_ 129.5) (Figure 2).

**Figure 3:**
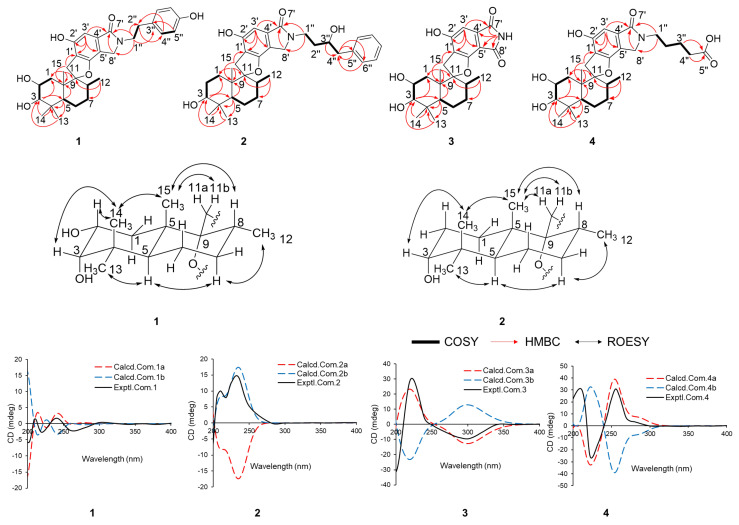
Key 2D NMR correlations, calculated and experimental CD spectra for compounds **1**–**4**.

The molecular formula of compound **2** was determined to be C_33_H_43_NO_5_. Its ^1^H and ^13^C NMR spectra were analogous to those of *N*-(2-benzenepropanoic acid) stachybotrylactam but displayed additional signals corresponding to a singly-substituted benzene ring and a 2-hydroxybutyl group spin system (Figures S9–S16). The connectivity of the 2-hydroxybutyl group from H-1″ to H-4″ was ascertained via ^1^H − ^1^H COSY correlations (Table 1, Figure 3 and Figure S15) [9]. Crucial HMBC correlations, such as those from H-1″ to C-7′ and C-8′ and from H-4″ to C-5″ and C-6″, ultimately confirmed the phenylspirodrimane skeleton and N-linked side-chain structure (Figure 1).

The molecular formula of **3** and **4** were determined to be C_23_H_29_NO_6_ and C_28_H_39_NO_7_ by HR-ESI-MS, respectively. A comparison of the ^1^H and ^13^C NMR data of **3** and **4** with those of **1** suggested that they contained the same C-2 hydroxylated phenylspirodrimane skeleton (Table 1 and Figures S17–S32). Besides the signals for the skeleton, only one additional N-H signal was observed at *δ*_H_ 8.39 for **3**, which showed HMBC correlations with C-8′ (*δ*_C_ 167.6), C-7′ (*δ*_C_ 169.5), C-4′ (*δ*_C_ 135.6), and C-5′ (*δ*_C_ 103.1), indicating the structure of **3** as shown in Figure 1. **4** contained additional N-linked signals for a valeric acid moiety (Table 1). The ^1^H-^1^H COSY correlations from H-1″ to H-4″ and the key HMBC correlations from H-1″ to C-7′ and C-8′ and from H-3″ and H-4″ to the carbonyl C-5″ confirmed the N-linked side chain (Table 1 and Figure 1).

The relative configurations of compounds **1**–**4** were determined by ROESY correlations (Figure 3). These results are in agreement with previous reports on PSDs-type compounds [4,9]. The experimental CD spectrum of compound **1** shows positive Cotton effects at 210 nm, 240 nm, and 300 nm and negative ones at 220 nm and 260 nm, which is consistent with the calculated ECD spectra of the most stable conformers of the enantiomer of 1b (2*R*, 3*S*, 5*S*, 8*R*, 9*R*, 10*S*) (Figure 3). Similarly, the absolute configurations of **2**, **3**, and **4** were assigned as 3*R*, 5*S*, 8*R*, 9*R*, 10*S* (2b), 2*R*, 3*S*, 5*S*, 8*R*, 9*R*, 10*S* (3a), and 2*R*, 3*S*, 5*S*, 8*R*, 9*R*, 10*S* (4a), respectively (Figure 3).

The nomenclature of PSDs is quite inconsistent. Terms like stachybotrysins, stachybotrins, stachybotrylactams, stachybotrylactones, dispirostachybotrylactams, and stachyflins are used [16]. Twenty PSDs were isolated from *Stachybotrys chartarum* and designated as chartarlactams A–T [4,9,15,17]. Following this naming convention, compounds **1**–**4** were named chartarlactams U–X.

**5**–**15** are known compounds, which are characterized by carefully comparing their NMR and HRMS data with those reported [4,6].

PSDs, a rare class of meroterpenoids (terpenylphenol) produced by fungi of the *Stachybotrys* genus, are characterized by the connection of a spirocyclic drimane and a phenyl moiety via a spirofuran ring. In our research, four novel compounds within this class were successfully isolated. Compared with the known compounds, compounds **1**, **3**, and **4** are distinct in that they possess an additional hydroxyl group at the 2-position of the phenylspirodrimane skeleton. On the other hand, compound **2** exhibits a unique feature: it has a novel N-substitution by a 3-hydroxy-4-phenylbutyl group. These newly isolated compounds expand our understanding of the structural diversity within the phenylspirodrimane family and may have the potential for further exploration in the fields of chemistry and biology.

### 2.2. Determination of Cytotoxicity and Inhibition of NO Production

**Figure 4:**
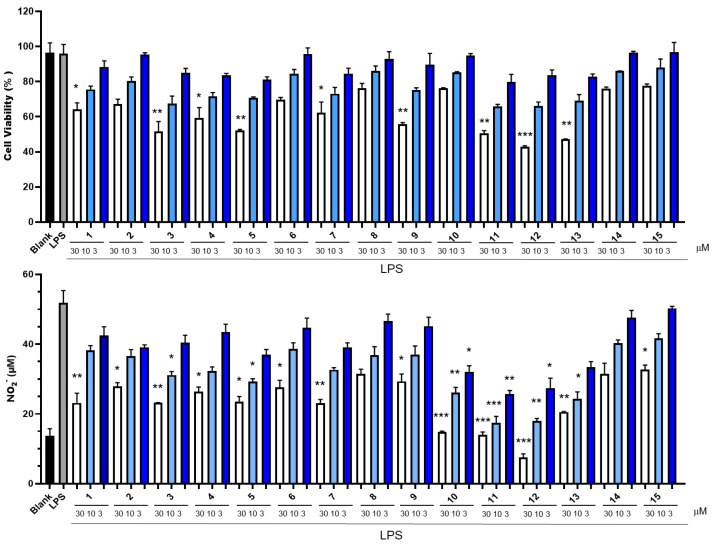
NO production inhibitory activities and cytotoxicities of compounds **1**–**15**. Values are the means ± SEM of 4 independent determinations. *, *p* < 0.05; **, *p* < 0.01; ***, *p* < 0.001 vs. LPS.

**Figure 5:**
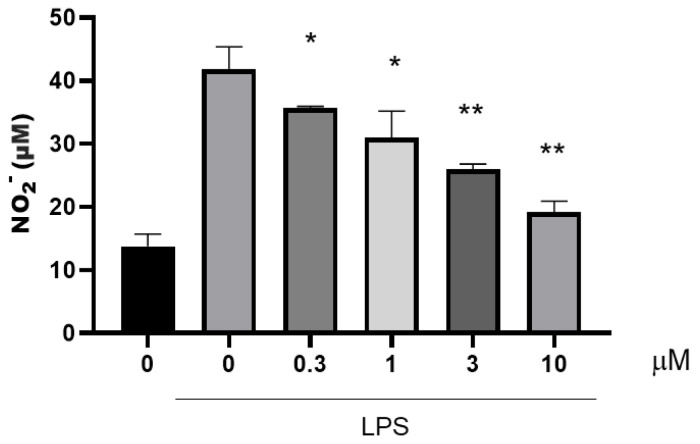
NO production inhibitory activities of compound **10** in RAW264.7 cells. Values are the means ± SEM of 4 independent determinations. *, *p* < 0.05; **, *p* < 0.01 vs. LPS.

The lipopolysaccharide (LPS)-stimulated mouse peritoneal macrophage (RAW 264.7) model was employed to assess the ability of all 15 compounds to suppress the generation of NO (Figure 4). The cytotoxicity of all compounds was tested at concentrations of 3 μM, 10 μM, and 30 μM. None of the compounds exhibited cytotoxicity at a concentration of 10 μM. Among these compounds, compound **10** exhibited the most potent inhibitory activity against NO production in the concentration range of 3 to 30 μM. It showed no detectable cytotoxicity, and its half-maximal inhibitory concentration (IC_50_) value was determined to be 12.4 μM. In contrast, although compounds **11** and **12** also displayed significant NO-inhibitory effects, they both manifested cytotoxicity at a concentration of 30 μM. Based on this, we conducted an in-depth study on the inhibitory activity of compound **10** against the release of inflammatory factors. We re-evaluated the inhibitory effect of compound **10** on NO production within the concentration range of 0.3–10 μM (Figure 5). Notably, even at a concentration as low as 0.3 μM, compound **10** demonstrated a potent inhibitory effect on NO production. This finding suggests its potential for the development of anti-inflammatory drugs.

### 2.3. Determination of the Ability of Compound ***10*** to Inhibit Inflammatory Cytokines

**Figure 6:**
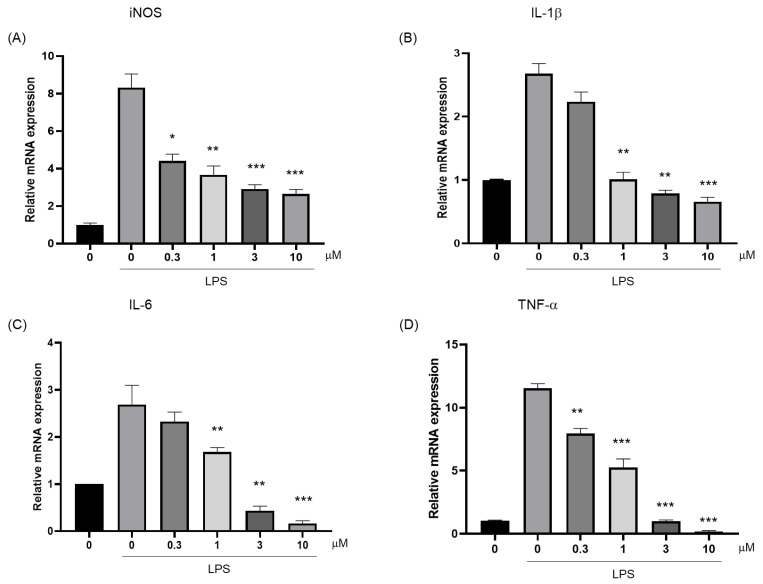
Inhibitory effects of LPS-induced inflammatory mediator expressions by Compound **10**. (**A**) Inhibitory effects of iNOS, (**B**) IL-1β, (**C**) IL-6, and (**D**) TNF-α expressions were measured by real time PCR. The RAW 264.7 cells were incubated for 24 h. *, *p* < 0.05; **, *p* < 0.01; ***, *p* < 0.001 vs. LPS.

NO is synthesized by inducible nitric oxide synthase (iNOS). In inflammation, interleukin-1β (IL-1β), interleukin-6 (IL-6), and tumor necrosis factor-α (TNF-α) can induce iNOS expression. In turn, NO produced by iNOS can also affect the production and activity of these cytokines, and they jointly regulate the inflammatory process. The anti-inflammatory efficacy of compound **10** was systematically evaluated through a quantitative analysis of critical inflammatory mediators. As shown in Figure 6A, the compound exhibited significant suppression of LPS-induced iNOS mRNA expression as assessed by RT-qPCR. Furthermore, compound **10** exerted potent inhibitory effects on proinflammatory cytokine production. A dose-dependent attenuation was observed in IL-1β (Figure 6B), IL-6 (Figure 6C), and TNF-α (Figure 6D) expression profiles.

### 2.4. Determination of the Inhibitory Effect of Compound ***10*** on the Binding Activity of Recombinant p65

Nuclear factor-kappa B (NF-κB), a master transcriptional regulator of inflammation, drives the expression of iNOS, IL-1β, IL-6, and TNF-α through κB consensus sequences in their promoter regions. Notably, these inflammatory mediators (e.g., NO and cytokines) reciprocally activate NF-κB via reactive oxygen species (ROS)-dependent signaling pathway, establishing a self-amplifying inflammatory cascade. Among NF-κB heterodimers, the p50–p65 complex predominates in the canonical signaling pathway, where the p65 subunit harbors a critical transactivation domain essential for recruiting transcriptional coactivators [23].

**Figure 7:**
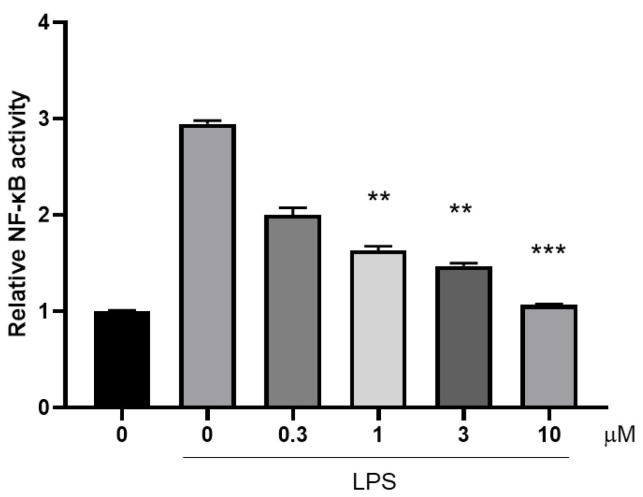
Inhibitory effects of recombinant p65 binding activity by compound **10**. **, *p* < 0.01; ***, *p* < 0.001.

To investigate the anti-inflammatory mechanism of compound **10**, we quantified its inhibitory effect on the DNA-binding activity of p65 using a TransAM^®^ NF-κB p65 ELISA kit (Ruixin Biotechnology, Guangzhou, China). Dose-response analysis revealed significant suppression of p65 binding at concentrations ranging from 0.3–10 μM (*p* < 0.01 vs. LPS-only group) (Figure 7).

### 2.5. Determination of the Ability of Compound ***10*** to Inhibit ROS

**Figure 8:**
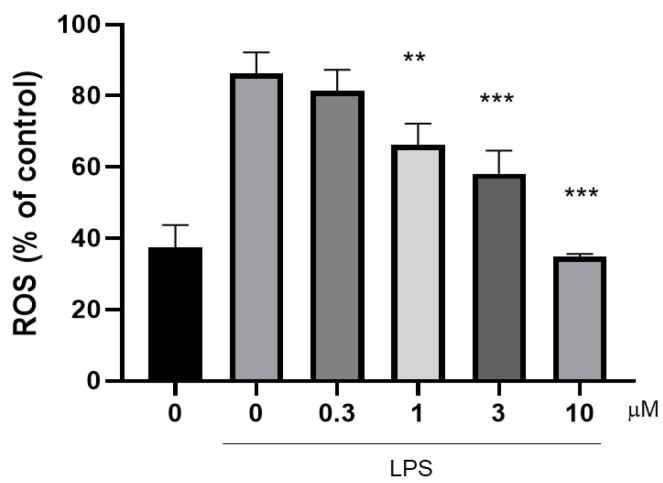
Inhibitory effects of LPS-induced ROS production by Compound **10**. The cells were incubated for 24 h. ROS production was measured by staining with the fluorescent probe. **, *p* < 0.01; ***, *p* < 0.001.

ROS and NF-κB have a complex relationship. During inflammation, stimuli such as TNF-α, LPS, and IL-1β can trigger the generation of ROS. ROS can activate NF-κB through multiple pathways. Conversely, activated NF-κB can not only inhibit ROS accumulation but also induce genes such as gp91phox, having antioxidant and pro-oxidative effects [24]. Compound **10** is capable of inhibiting the production of ROS at concentrations that are not cytotoxic (Figure 8), which suggests that compound **10** may inhibit the activity of NF-κB by inhibiting the accumulation of ROS, thereby producing an anti-inflammatory effect [22].

## 3. Discussion

In the present study, 15 PSDs were isolated from *Stachybotrys* sp. SZU-W23 and their structures were elucidated. Among them, four novel compounds, namely chartarlactams U–W (**1**–**4**), were identified. These results not only expand the PSDs family but also enrich the structural diversity of natural products derived from *Stachybotrys* fungi. The anti-inflammatory activity evaluation using the LPS-induced RAW 264.7 cell model revealed that several compounds, especially compound **10**, exhibited significant anti-inflammatory effects with an IC_50_ values of 12.4 μM. Three PSDs-type compounds, namely stachybotrysin C, stachybonoid F, and stachybotrylactone, have been reported to exhibit moderate anti-inflammatory activity [25]. This activity was demonstrated by their ability to inhibit NO production in LPS-activated RAW264.7 murine macrophages, with IC_50_ values of 27.2 μM, 52.5 μM, and 17.9 μM, respectively. However, the molecular mechanisms underlying this anti-inflammatory action remain largely uncharacterized. As such, both the anti-inflammatory efficacy of PSDs and the intricate biological pathways mediating these effects warrant comprehensive investigation. The inhibitory effect of compound **10** on iNOS, IL-6, and IL-1β expression aligns with the known anti-inflammatory mechanisms of related compounds that target key mediators in the inflammatory cascade.

Our results suggest that for PSDs, the side chains, rather than the main skeleton, are the primary determinants of cytotoxicity, as exemplified by compounds **14** and **15** with low cytotoxicity. The 2-position hydroxyl group was found to increase cytotoxicity, as demonstrated by the compound pairs 4/5, 8/9, and 11/12.

Regarding NO production inhibition, the N-side chain substituents significantly influenced activity. At a test concentration of 10 μM, compounds **10**, **11**, and **12**, which feature an N-side chain isovaleric acid substitution, exhibited potent inhibitory effects with IC_50_ values of 12.4, 4.4, and 6.5 μM, respectively. Compound **13** (IC_50_ = 11.1 μM) also showed good activity, likely attributed to its dual carboxyl side chain. Compound **1** (IC_50_ = 26.2 μM), with a phenolic side chain, and compound **3** (IC_50_ = 22.4 μM), which has an 8′-carbonyl on the main skeleton, demonstrated moderate and good inhibitory effects, respectively, indicating that these functional groups contribute to enhanced NO inhibition.

Mechanistically, the ability of compound **10** to inhibit the binding activity of p65 and the production of ROS suggests a complex interplay within the NF-κB/ROS pathway [23]. Inflammation is a highly regulated process, and the NF-κB pathway is a central player, controlling the transcription of numerous inflammation-related genes. ROS can act as signaling molecules, either promoting or inhibiting NF-κB activation depending on the cellular context. Compound **10** might disrupt this complex network by suppressing ROS production, which in turn inhibits NF-κB activation, leading to a reduction in the expression of iNOS and pro-inflammatory cytokines [26]. This anti-inflammatory mechanism aligns with the current understanding of how natural products modulate the inflammatory response at the molecular level.

However, several aspects of this study warrant further exploration. First, the in vivo anti-inflammatory effects of these compounds remain to be investigated. Although the in vitro experiments demonstrated promising anti-inflammatory activities, the complex physiological environment in vivo may yield different outcomes. Factors such as metabolism, distribution, and interaction with other biological molecules could influence the efficacy and safety of these compounds. Second, the precise molecular interactions of compound **10** with the NF-κB/ROS pathway components need to be elucidated. For instance, it is unclear whether compound **10** directly binds to specific proteins in the pathway or acts via an indirect mechanism. Advanced techniques, such as protein-protein interaction assays and molecular docking studies, could provide more in-depth insights into these interactions.

## 4. Materials and Methods

### 4.1. Fungal Strain

*Stachybotrys* sp. SZU-W23 was collected from the bottom sediments of the intertidal zone of Shenzhen and deposited in the Marine Microbial Lab of the College of Life Sciences and Oceanography, Shenzhen University (Shenzhen, China).

### 4.2. General Experimental Procedures

The NMR spectra were acquired on a Bruker ASCEND 600 MHz NMR magnet system (Bruker Biospin GmbH, Rheinstetten, Germany) equipped with a CryoProbe, and TMS was used as the internal standard. HR-ESI-MS was carried out using a MaXis quadrupole-time-of-flight mass spectrometer (Bruker Biospin GmbH, Rheinstetten, Germany). Column chromatography was performed using normal-phase silica gel (200–300 mesh, Qingdao Haiyang Chemical Factory, Qingdao, China) and reversed-phase ODS-A (150 µM, YMC Co., Ltd., Kyoto, Japan). HPLC was performed on a Shimadzu LC-16P system (Shimadzu Co., Tokyo, Japan) fitted with a YMC-Pack ODS-A C18 column (20 × 250 mm, 5 µm; YMC Co., Ltd., Kyoto, Japan). Analytical-grade and HPLC-grade reagents (Macklin Co., Shanghai, China) were utilized for the isolation procedures. Optical rotations were measured on a Jasco J-815 polarimeter (Jasco, Tokyo, Japan).

### 4.3. Fermentation, Extraction, and Isolation

Sucrose 5 g, powdered yeast extract 0.1 g, NaNO_3_ 0.3 g, K_2_HPO_4_ 0.1 g, MgSO_4_·7H_2_O 0.05 g, KCl 0.05 g, CoCl_2_·6H_2_O 0.00025 g, FeSO_4_·7H_2_O 0.0015 g, and CaCl_2_·2H_2_O 0.00065 g were dissolved in 100 mL of deionized water. The pH was adjusted to 5.8 with HCl. Sterilization was performed at 121 °C for 30 min. After 96 h fermentation (on day 4), 100 mg of ammonium chloride was added to the medium and the culture was continued. Ethanol was added to terminate the fermentation five days later.

A 10 L fermentation broth was finally extracted with ethyl acetate to afford 1.97 g of crude extract.

The crude extract was subjected to a silica gel column, yielding 10 fractions. Fractions 7, 8, and 9 were pooled together, yielding 394.6 mg of the combined material. Subsequently, column chromatography was carried out on this combined fraction using a Sephadex LH-20 gel column (2 cm × 90 cm) with 40% methanol (MeOH) as the eluent, resulting in the isolation of 14 subfractions. Then, subfractions 8, 9, 11, and 12 were further isolated by high performance liquid chromatography (HPLC) (20 × 250 mm, 10 mL/min). A gradient elution was carried out with 70–75% of 0.1% formic acid in acetonitrile for 15 min at a flow rate of 1 mL/min. Compounds **1** and **2** were obtained at 9.5 min and 15.0 min, respectively, from subfraction 11. Compounds **3**, **4**, **5**, and **6** were obtained at 11.5 min, 12.5 min, 13.5 min, 14.5 min, and 15.5 min, respectively, from subfraction 12. Compounds **7**, **10**, **11**, **12**, and **13** were obtained at 8.0 min, 8.5 min, 10.0 min, 10.5 min, and 12.5 min, respectively, from subfraction 8. Compounds **8**, **9**, **14**, and **15** were obtained at 11.0 min, 11.5 min, 12.5 min, and 15.0 min, respectively, from sub-fraction 9.

### 4.4. Characterization of Novel Compounds

*Chartarlactam U*. white powder; UV (MeOH) λmax (log ε) 229 (4.56) nm; ^1^H-NMR and ^13^C-NMR data, see Table 1; HRESIMS *m*/*z* 522.2879 [M + H]^+^ (calcd for C_31_H_40_NO_6_, 522.2850), *m*/*z* 1043.5576 [2M + H]^+^ (calcd for C_62_H_79_N_2_O_12_, 1043.5560).

*Chartarlactam V.* white powder; UV (MeOH) λmax (log ε) 225 (4.54) nm; ^1^H-NMR and ^13^C-NMR data, see Table 1; HRESIMS *m/z* 534.3275 [M + H]^+^ (calcd for C_33_H_44_NO_5_, 534.3276), *m*/*z* 556.2137 [M + Na]^+^ (calcd for C_33_H_43_NNaO_5_, 556.2144), *m*/*z* 1089.3300 [2M + Na]^+^ (calcd for C_66_H_86_N_2_NaO_10_, 1089.3305).

*Chartarlactam W.* white powder; UV (MeOH) λmax (log ε) 224 (4.54) nm; ^1^H-NMR and ^13^C-NMR data, see Table 1; HRESIMS *m/z* 438.1906 [M + Na]^+^ (calcd for C_23_H_29_NNaO_6_, 438.1887), *m*/*z* 853.3860 [2M + Na]^+^ (calcd for C_46_H_58_N_2_NaO_12_, 853.3884).

*Chartarlactam X.* white powder; UV (MeOH) λmax (log ε) 215 (4.51) nm; ^1^H-NMR and ^13^C-NMR data, see Table 1; HRESIMS *m*/*z* 502.2804 [M + H]^+^ (calcd for C_28_H_40_NO_7_, 502.2811), *m*/*z* 524.2610 [M + Na]^+^ (calcd for C_28_H_39_NNaO_7_, 524.2622).

### 4.5. Cell Culture

The murine macrophage cell line RAW 264.7 was obtained from the American Type Culture Collection (ATCC, Manassas, VA, USA). The cells were cultured in high-glucose Dulbecco’s Modified Eagle Medium (DMEM) supplemented with 10% (*v*/*v*) heat-inactivated fetal bovine serum (Qingmu Bio, Wuhan, China), 100 U/mL penicillin, and 100 μg/mL streptomycin. Cultures were incubated at 37 °C in a humidified atmosphere containing 5% CO_2_.

### 4.6. CCK8 Assay

RAW 264.7 cells were seeded into 96-well plates at a density of 1.0 × 10^5^ cells/well (100 μL/well) in complete DMEM (high-glucose DMEM supplemented with 10% FBS, 100 U/mL penicillin, and 100 μg/mL streptomycin) and allowed to adhere for 24 h at 37 °C under 5% CO_2_. Subsequently, serial dilutions of compounds (0.3–30 μM in DMSO, with the final DMSO concentration < 0.1%) were added to respective wells and incubated for 24 h. Cell viability was assessed using the CCK-8 assay (Dojindo Laboratories, Kumamoto, Japan): 10% (*v*/*v*) CCK-8 reagent was added to each well, followed by incubation for 30 min at 37 °C. The absorbance was measured at 450 nm with a reference wavelength of 650 nm using a microplate reader (BioTek Instruments, Winooski, VT, USA).

### 4.7. Measurement of NO Production

RAW 264.7 macrophages were seeded into 96-well plates at a density of 2.0 × 10^5^ cells/well in complete DMEM and allowed to adhere for 2 h at 37 °C under 5% CO_2_. The cells were pretreated with compounds **1**–**15** (3–30 μM) for 2 h, followed by stimulation with ultrapure lipopolysaccharide (LPS; 100 ng/mL, from *E. coli* O111:B4, Sigma-Aldrich) for 24 h. NO production was assessed via the Griess reaction by quantifying nitrite (NO_2_^−^) accumulation in culture supernatants.

Briefly, 50 μL of the cell-free supernatant from each well was mixed with 50 μL of 1% sulfanilamide (dissolved in 5% phosphoric acid; Griess Reagent A, Beyotime Biotechnology, Shanghai, China) and incubated for 5 min at room temperature. Subsequently, 50 μL of 0.1% N-1-naphthylethylenediamine dihydrochloride (Griess Reagent B, Beyotime Biotechnology) was added, followed by 5 min incubation in the dark. The absorbance was measured at 540 nm with a reference wavelength of 630 nm using a Synergy H1 microplate reader (BioTek Instruments, Winooski, VT, USA). Nitrite concentrations were interpolated from a sodium nitrite standard curve (0–100 μM; R^2^ > 0.99).

### 4.8. RNA Isolation and Real-Time PCR

RAW 264.7 cells (2 × 10^6^ cells) were seeded into 30 mm petri dishes. After the cells adhered to the dishes for 2 h, compound **10** at concentrations of 0.3, 1, 3, and 10 μM was added, and the cells were cultured in an incubator at 37 °C for 2 h. Then, 100 ng/mL of LPS was added and the cells were co-cultured for 24 h. The cells were collected into 1.5 mL EP tubes, and total RNA was extracted using an RNA extraction kit. Then, the concentration of the total RNA was determined with a nucleic acid quantifier, and the RNA was reverse transcribed into the corresponding cDNA.

According to the operation procedures of the rapid reverse transcription kit, 1 μg of total RNA and 1 μL of dNTP Mixture were first added into a PCR tube. Then, RNase-free dH_2_O was added until the total volume of the solution reached 10 μL. After mixing evenly, the mixture was incubated at 65 °C for 5 min and then immediately placed on ice for ice-bathing. Subsequently, 4 μL of 5 × PrimeScript^®^ Buffer, 0.5 μL of RNase Inhibitor (40 U/μL), 1 μL of PrimeScript^®^ RTase (200 U/μL), and 4.5 μL of RNase Free dH_2_O were added to make the entire solution system reach 20 μL. After mixing the solution evenly, it was placed in a PCR instrument, and the parameters were set as 30 °C for 10 min, 45 °C for 45 min, 70 °C for 15 min, 95 °C for 5 min, and 4 °C for 5 min, finally obtaining the cDNA.

The reaction system mixture was prepared using a real-time quantitative PCR kit. First, 1 μL of the first-strand cDNA was added to 14 μL of KOD FX Neo PCR buffer and dNTPs (Toyobo Co., LTD., Osaka, Japan). Then, 1 μL of the primers was added, and the amplification was performed with the following conditions: 95 °C for 60 s, followed by 40 cycles of 95 °C for 15 s, 60 °C for 15 s, and 72 °C for 45 s, using a Stratagene Mx3000P (Aglient Technologies Inc., Palo Alto, CA, USA). The following primer pairs were used: IL-1β sense, 5′-CGTGGACCTTCCAGGATGAG-3′, antisense, 5′-GGAGCCTGTAGTGCAGCTGTC-3′; IL-6 sense, 5′-ACCACGGCCTTCCCTACTTC-3′, antisense, 5′-CACAACTCTTTTCTCATTTCCACG-3′; iNOS sense, 5′-GCAGCTACTGGGTCAAAGACAA-3′, antisense, 5′-TCTCTGCCTATCCGTCTCGTC-3′; TNF-α sense, 5′-GACCCTCACACTCAGATCATCTTCT-3′, antisense, 5′-CCTCCACTTGGTGGTTTGCT-3′ and GAPDH sense, 5′-TGCACCACCAACTGCTTAG-3′, antisense, 5′-GATGCAGGGATGATGTTC-3′.

### 4.9. Measurement of Binding Activity of p65

RAW 264.7 macrophages were seeded into 60 mm diameter culture dishes at a density of 3.0 × 10^6^ cells/dish in complete DMEM and allowed to adhere for 2 h at 37 °C under 5% CO_2_. Cells were pretreated with Compound **10** (0.3–10 μM in DMSO, final solvent concentration < 0.1%) for 2 h, followed by stimulation with ultrapure LPS for an additional 2 h. The supernatant of cell culture medium was collected, and then NF-κB p65 binding activity of NF-κB in extracts was detected with NF-κB p65 kit (RUIXIN, Quanzhou, China).

### 4.10. Measurement of Reactive Oxygen Species (ROS) Production

RAW264.7 cells were seeded into black 96-well plates at a density of 5 × 10^4^ cells per well and cultured for 2 h until they adhered to the plate. Subsequently, compound **10** at concentrations of 0.3, 1, 3, and 10 μM was added respectively, and the cells were cultured for another 2 h. Then, 100 ng/mL of LPS was added and the cells were co-cultured in an incubator at 37 °C for 24 h. After that, the production of ROS was detected. The cell culture medium in the 96-well plates was removed, and then 1 mL of the fluorescent probe DCFH-DA solution diluted to 10 μM with serum-free DMEM medium was added. The plates were placed in the cell incubator and incubated in the dark for 30 min. Subsequently, the supernatant was removed and the cells were washed three times with phosphate-buffered saline (PBS). Finally, detection was carried out using a microplate reader (BioTek, Winooski, VT, USA) with an excitation wavelength of 488 nm and an emission wavelength of 525 nm.

### 4.11. Statistical Analysis

Statistical comparison was carried out using one-way ANOVA and/or the Student’s *t*-test. Statistical analysis was performed using GraphPad Prism 8 software (San Diego, CA, USA), and the results were presented as the mean ± SEM.

## 5. Conclusions

In conclusion, this investigation systematically explored the anti-inflammatory potential of PSDs synthesized by the marine-derived fungal strain *Stachybotrys* sp. SZU-W23. A total of 15 PSDs were successfully isolated, among which four novel compounds, chartarlactams (U-X), were precisely characterized. Significantly, compound **10** exhibited the most prominent inhibitory effect on the overproduction of NO in LPS-stimulated RAW 264.7 macrophages. Mechanistic studies showed that compound **10** effectively suppressed the generation of ROS, which might in turn inactivate the NF-κB signaling pathway and consequently downregulate pro-inflammatory mediators such as iNOS, IL-6, and IL-1β. These findings not only enrich our understanding of the bioactive compounds from marine-derived fungi but also highlight the potential of PSDs as a source for developing novel anti-inflammatory agents.

## Figures and Tables

**Table 1:** ^1^H NMR and ^13^C NMR Data for Compounds **1**–**4** in CDCl_3_.

		1		2		3		4
No.	*δ*_C_ *^a^*	*δ*_H_ (*J* in Hz) *^b^*	*δ*_C_ *^a^*	*δ*_H_ (*J* in Hz) *^b^*	*δ*_C_ *^a^*	*δ*_H_ (*J* in Hz) *^b^*	*δ*_C_ *^a^*	*δ*_H_ (*J* in Hz) *^b^*
1	33.3, CH_2_	1.60, t (12.0)	24.3, CH_2_	0.93, m *^c^*	33.3, CH_2_	1.57, t (11.7)	33.3, CH_2_	1.63, m
		1.10, dd (12.0, 4.4)		1.79, m *^c^*		1.14, dd (11.7, 4.4)		1.12, dd (11.9, 4.3)
2	65.3, CH	3.81, m *^c^*	25.3, CH_2_	1.79, m *^c^*	65.3, CH	3.81, m *^c^*	65.3, CH	3.82, br d (12.1)
				1.41, m *^c^*				
3	78.1, CH	3.14, brs	73.9, CH	3.20, brs	77.9, CH	3.16, brs	78.1, CH	3.16, m *^c^*
4	38.6, C		37.8, C		38.6, C		38.6, C	
5	39.2, CH	1.92, dd (12.5, 4.0)	39.8, CH	2.04, dd (12.1, 3.1)	39.2, CH	1.97, dd (12.5, 3.1)	39.2, CH	1.95, dd (12.5, 3.0)
6	20.7, CH_2_	1.46, m *^c^*	20.9, CH_2_	1.48, m *^c^*	20.7, CH_2_	1.49, m *^c^*	20.7, CH_2_	1.49, m *^c^*
		1.36, m *^c^*		1.42, m *^c^*		1.42, m *^c^*		1.38, m *^c^*
7	31.2, CH_2_	1.53, m *^c^*	31.2, CH_2_	1.53, m *^c^*	31.4, CH_2_	1.55, m *^c^*	31.2, CH_2_	1.54, m *^c^*
		1.36, m *^c^*		1.42, m *^c^*		1.40, m *^c^*		1.38, m *^c^*
8	36.6, CH	1.78, m *^c^*	37.0, CH	1.77, m *^c^*	36.7, CH	1.84, m *^c^*	36.6, CH	1.80, m *^c^*
9	98.1, C		98.2, C		100.4, C		98.1, C	
10	43.4, C		42.2, C		43.3, C		43.4, C	
11	32.3, CH_2_	3.13, d (14.2)	32.2, CH_2_	3.11, d (16.7)	30.9, CH_2_	3.11, d (17.2)	32.3, CH_2_	3.15, d (16.9)
		2.76, d (14.2)		2.72, d (16.7)		2.76, d (17.2)		2.78, d (16.9)
12	15.9, CH_3_	0.64, d (6.4)	16.0, CH_3_	0.60, d (6.5)	15.8, CH_3_	0.64, d (6.5)	15.9, CH_3_	0.66, d (6.5)
13	29.4, CH_3_	0.92, s	29.1, CH_3_	0.90, s	29.4, CH_3_	0.96, s	29.4, CH_3_	0.94, s
14	22.5, CH_3_	0.81, s	22.8, CH_3_	0.81, s	22.5, CH_3_	0.84, s	22.5, CH_3_	0.83, s
15	17.2, CH_3_	0.97, s	16.3, CH_3_	0.95, s	17.3, CH_3_	0.99, s	17.2, CH_3_	0.99, s
1′	116.8, C		116.5, C		119.8, C		116.7, C	
2′	154.2, C		153.9, C		159.7, C		154.2, C	
3′	101.3, CH	6.55, s	101.1, CH	6.46, s	104.0, CH	6.67, s	101.3, CH	6.57, s
4′	134.6, C		134.7, C		135.6, C		134.6, C	
5′	112.4, C		112.8, C		103.1, C		112.4, C	
6′	156.2, C		156.2, C		158.7, C		156.3, C	
7′	167.7, C		168.2, C		169.5, C		167.9, C	
8′	47.4, CH_2_	4.26, d (16.8)	44.6, CH_2_	4.51, d (16.4)	167.6, C		47.0, CH_2_	4.30, d (16.8)
		4.20, d (16.8)		4.11, d (16.4)				4.19, d (16.8)
1″	44.1, CH_2_	3.61, t (7.0)	40.7, CH_2_	4.15, brs			41.7, CH_2_	3.47, t (7.2)
				2.40, brs				
2″	33.6, CH_2_	2.79, m *^c^*	29.5, CH_2_	2.63, brs			27.7, CH_2_	1.62, m *^c^*
				1.25, brs				
3″	129.5, C		56.9, CH	4.85, brs			22.4, CH_2_	1.46, m *^c^*
4″	129.9, CH	7.04, d (7.0)	36.3, CH_2_	3.42, brs			33.8, CH_2_	2.25, t (7.4)
				3.06, m				
5″	115.6, CH	6.67, d (7.0)	140.1, C				175.0, C	
6″	156.2, C		128.6, CH	7.23, m *^c^*				
7″			128.9, CH	7.20, m *^c^*				
8″			126.3, CH	7.10, t (7.1)				
2′-OH						0.75, s		9.84, s
2-OH						4.18, brs		
3-OH						3.99, d (3.3)		
NH						8.39, brs		

*^a^* 150 MHz. *^b^* 600 MHz. *^c^* Overlapped signals.

## Data Availability

The original data presented in the study are included in the article/Supplementary Material.

## Supplementary Materials

The following supporting information can be downloaded at: https://www.mdpi.com/article/10.3390/md23050216/s1, Figures S1–S32 present the NMR spectra of compounds **1**–**4**. Meanwhile, Tables S1–S4 list the coordinates (in Ångstroms) of the conformers for compounds **1**–**4**.
