# Supplementary material for: Chartarlactams U-X: Novel Phenylspirodrimanes from a Marine Derived Fungus Stachybotrys sp. SZU-W23 with Anti-Inflammatory Activity Mediated by the NF-κB/ROS Signaling Pathways
Source: Mar Drugs. 2025 May 20;23(5):216. doi: 10.3390/md23050216 (PMC12113431; doi:10.3390/md23050216)
Supplement: Supplementary file 1 [file marinedrugs-23-00216-s001.zip › marinedrugs-3616690-supplementary.pdf]

marine drugs

Article

Supplementary Information

Chartarlactams U-X: Novel Phenylspirodrimanes from a Marine Derived Fungus *Stachybotrys* sp. SZU-W23 with Anti-Inflammatory Activity Mediated by the NF-κB/ROS signaling pathways

Yanhua Wu [1,2], Lanyi Lu [1], Peng Zhang [3,*] and Liyan Wang [1,*]

1 Shenzhen Key Laboratory of Microbial Genetic Engineering, College of Life Sciences and Oceanography, Shenzhen University, Shenzhen 518060, China; wuyanhua@szu.edu.cn (Y.W.); 2200251008@email.szu.edu.cn (L.L.)
2 College of Physics and Optoelectronic Engineering, Shenzhen University, Shenzhen 518060, China
3 State Key Laboratory of Discovery and Utilization of Functional Components in Traditional Chinese Medicine, Natural Products Research Center of Guizhou Province, Guiyang 550014, China
* Correspondence: lwang@szu.edu.cn (L.W.); 15720389@stu.ahau.edu.cn (P.Z.)

Academic Editors: Bae Munhyung, Jae-hyuk Jang

Received: 15 April 2025
Revised: 11 May 2025
Accepted: 16 May 2025
Published: 20 May 2025

Citation: Wu, Y.; Lu, L.; Zhang, P.; Wang, L. Chartarlactams U-X: Novel Phenylspirodrimanes from a Marine Derived Fungus *Stachybotrys* sp. SZU-W23 with Anti-Inflammatory Activity Mediated by the NF-κB/ROS Signaling Pathways. *Mar. Drugs* **2025**, *23*, 216. https://doi.org/10.3390/md23050216

Copyright: © 2025 by the author. Licensee MDPI, Basel, Switzerland. This article is an open access article distributed under the terms and conditions of the Creative Commons Attribution (CC BY) license (https://creativecommons.org/licenses/by/4.0/).

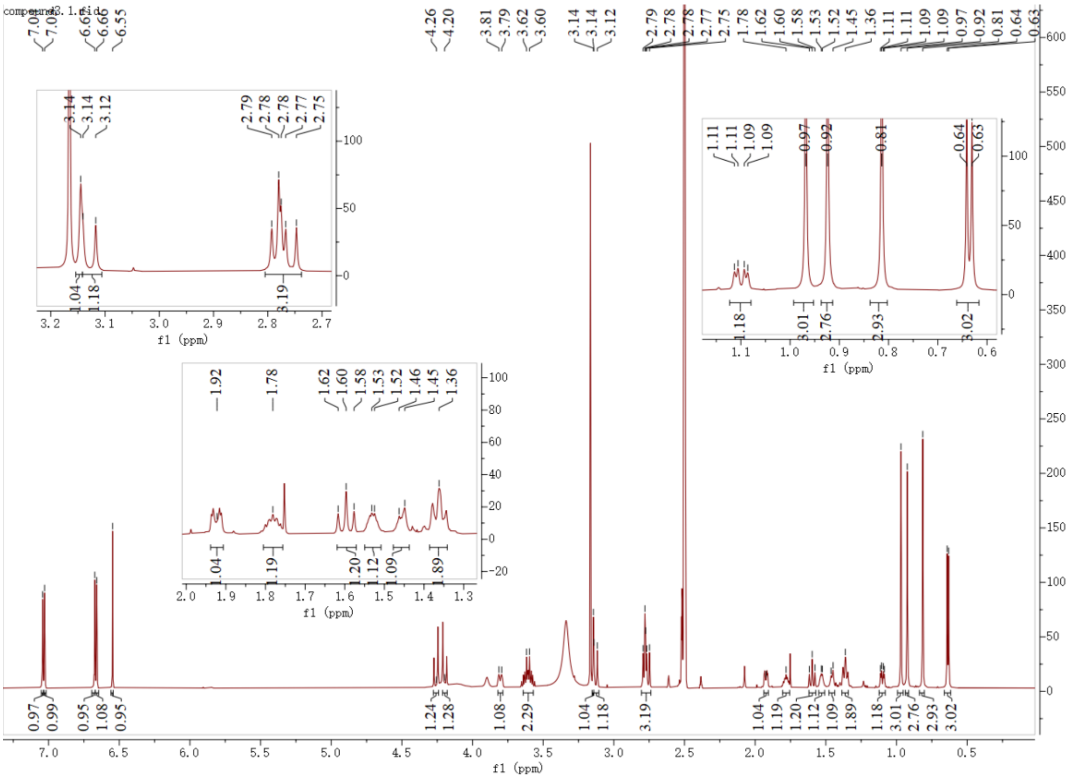

Figure S1. ^{1}H NMR spectrum of compound **1** (in DMSO-d_6; δ, ppm; *J*, Hz).

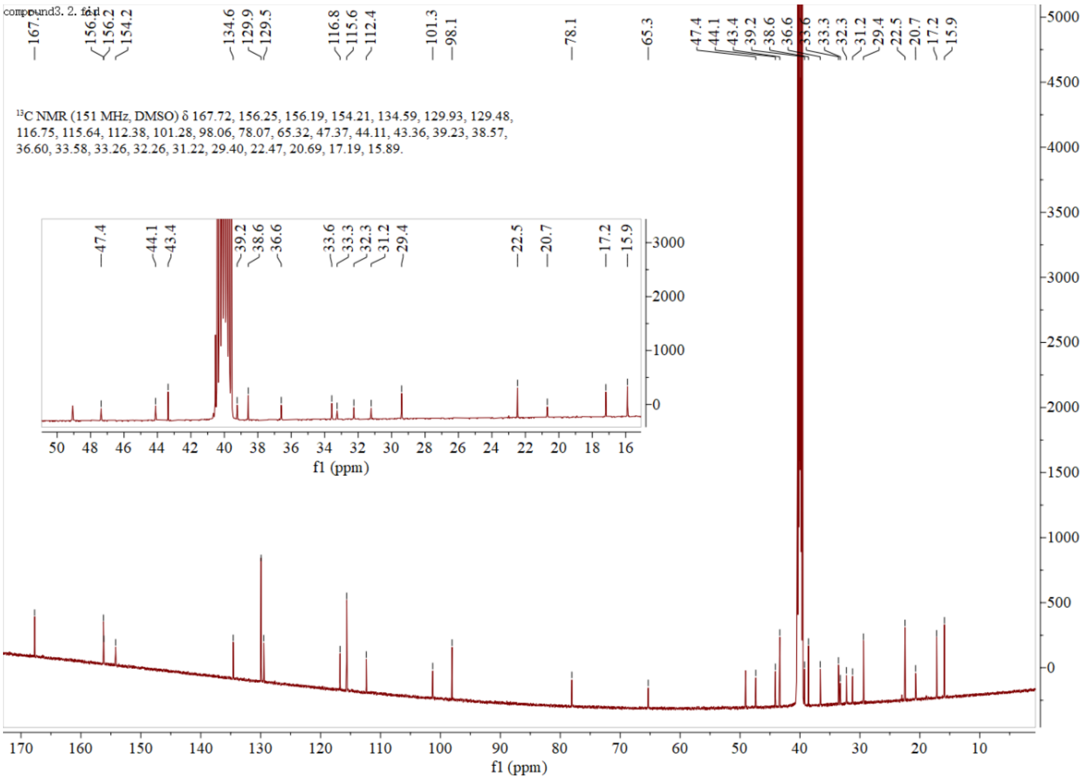

Figure S2. ^{13}C NMR spectrum of compound **1** (in DMSO-d_6; δ, ppm).

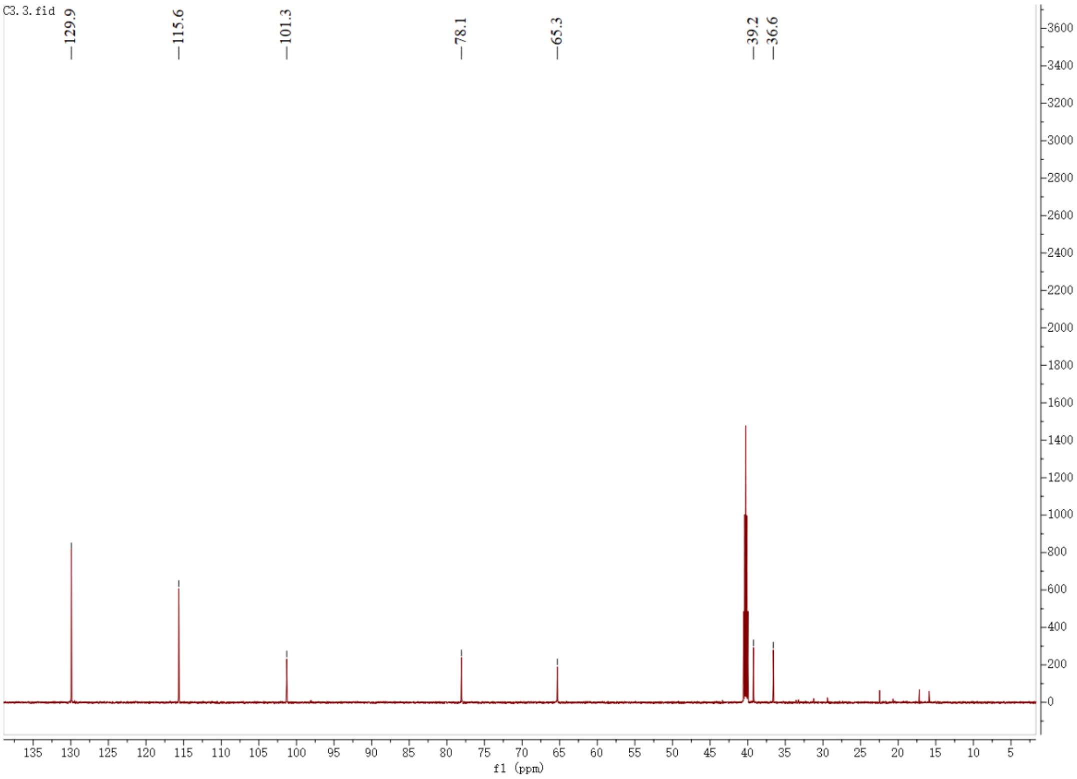

Figure S3. DEPT-90 spectrum of compound **1** (in DMSO-d_6; δ, ppm).

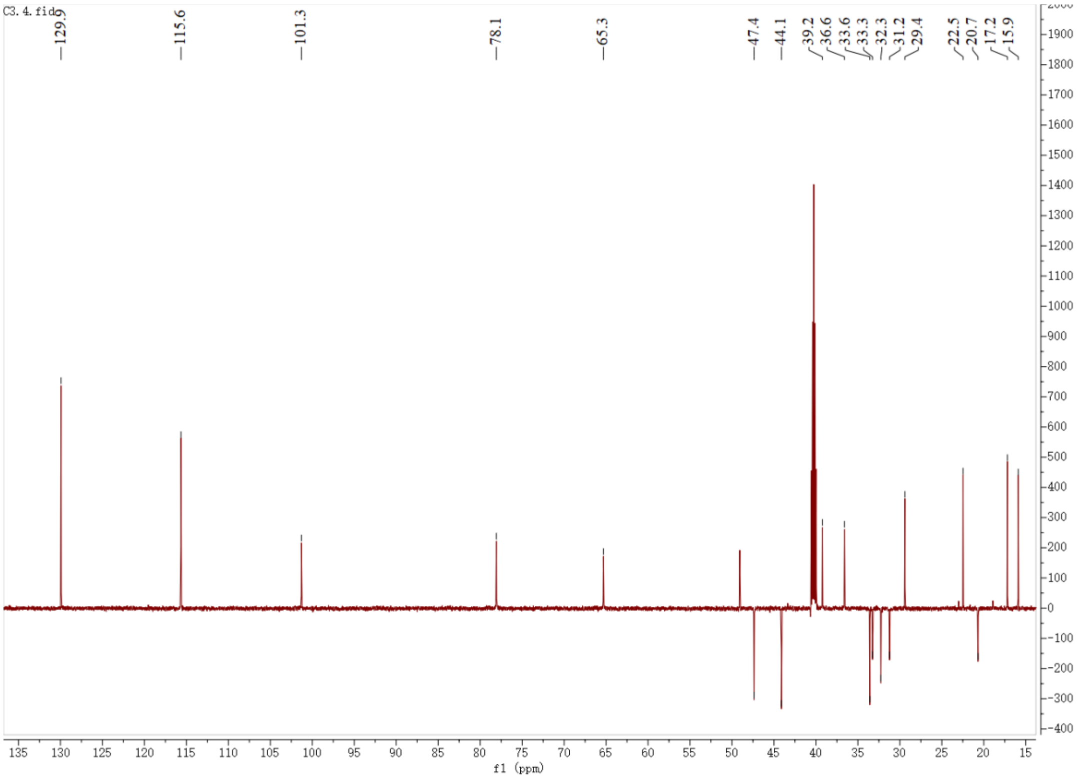

Figure S4. DEPT–115 spectrum of compound **1** (in DMSO-d_6; δ, ppm).

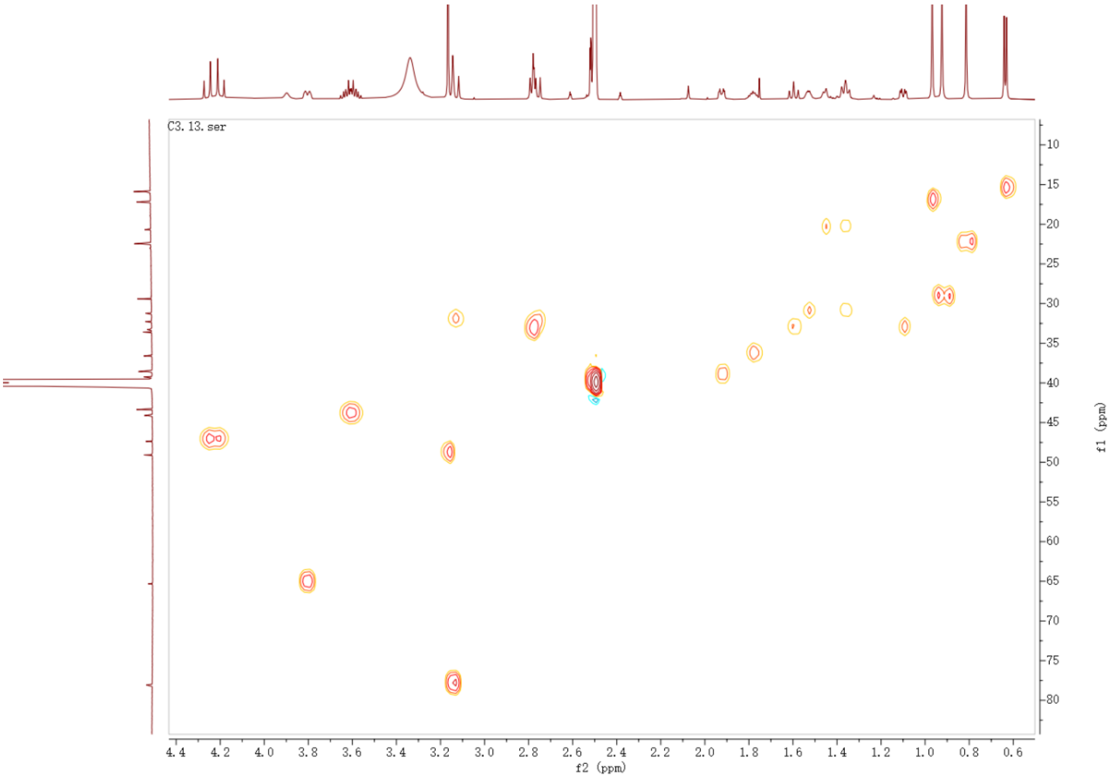

Figure S5. HMQC spectrum of compound **1** (in DMSO-d_6; δ, ppm).

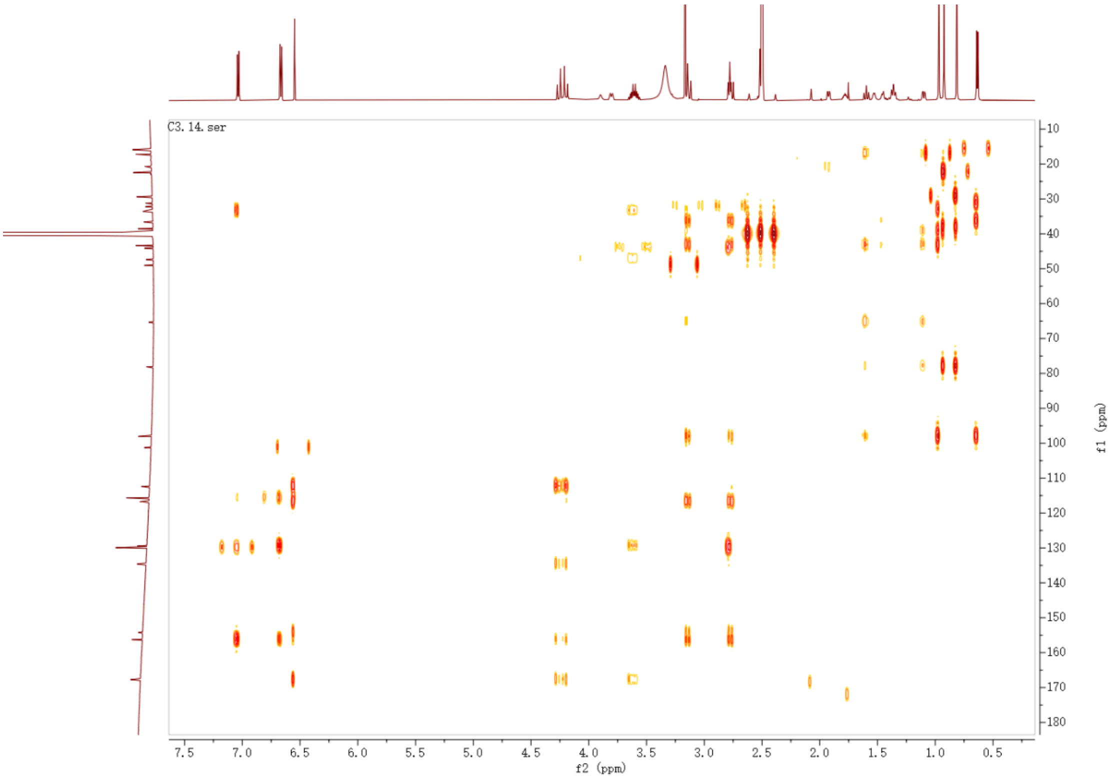

Figure S6. HMBC spectrum of compound **1** (in DMSO-d_6; δ, ppm).

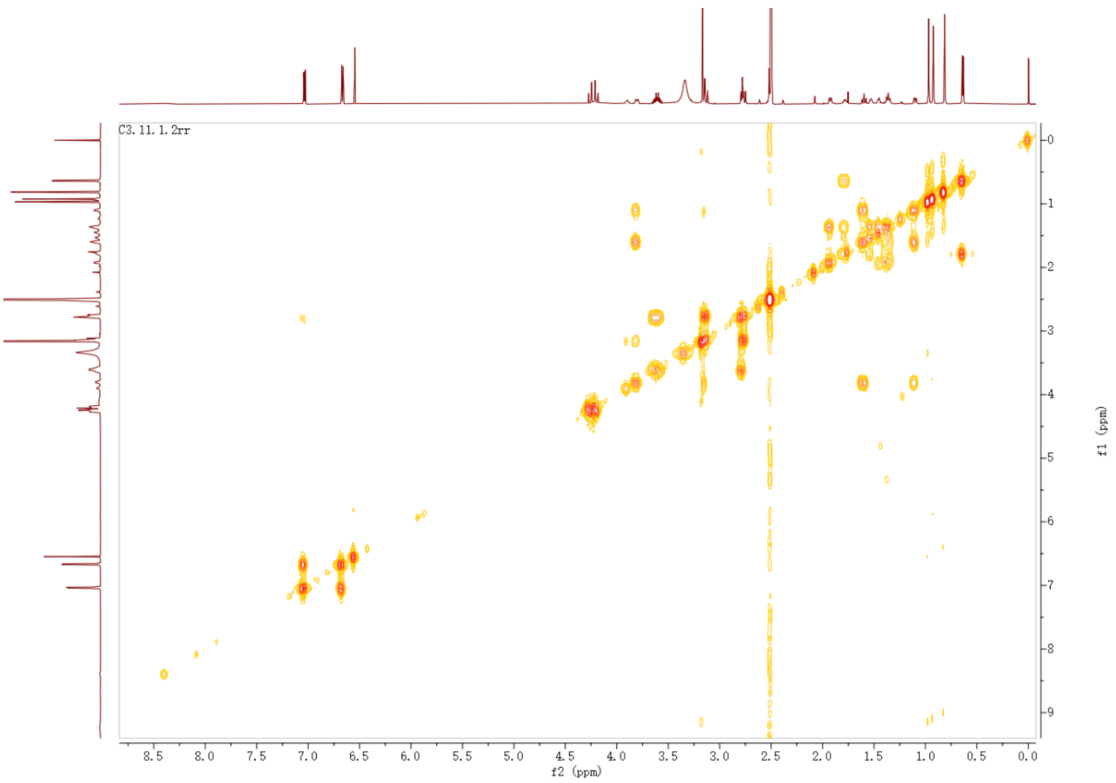

Figure S7. ^{1}H–^{1}H COSY spectrum of compound **1** (in DMSO-d_6; δ, ppm).

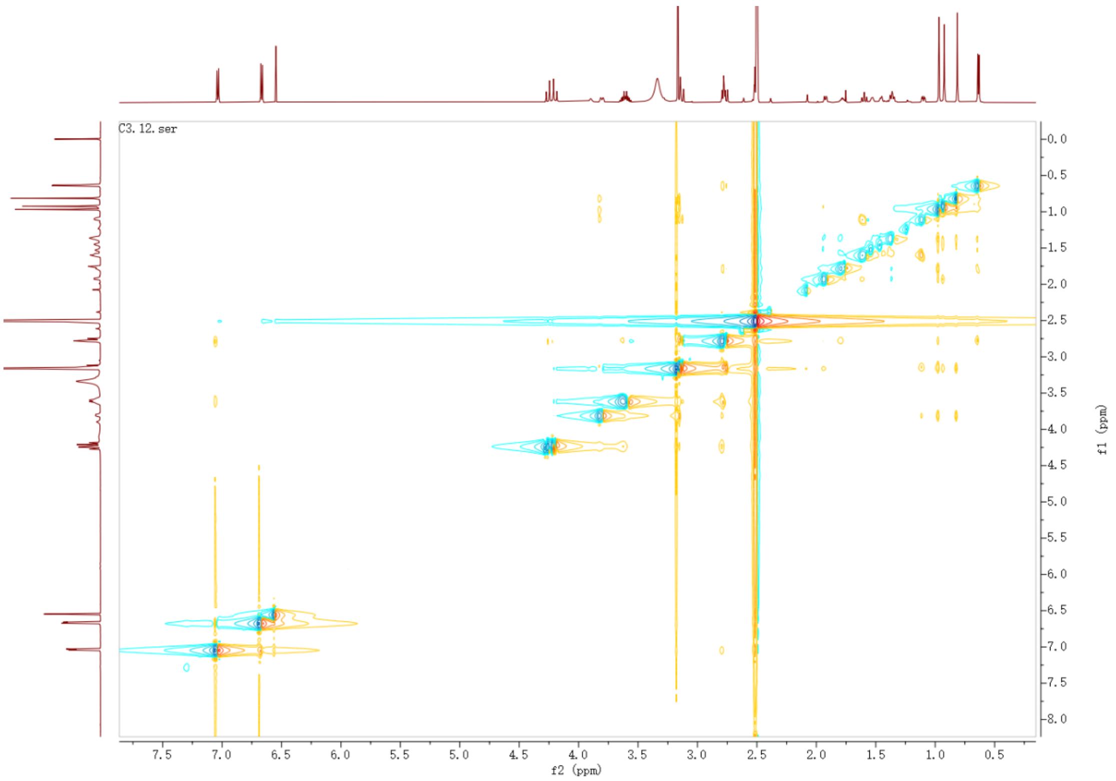

Figure S8. ROESY spectrum of compound **1** (in DMSO-d_6; δ, ppm).

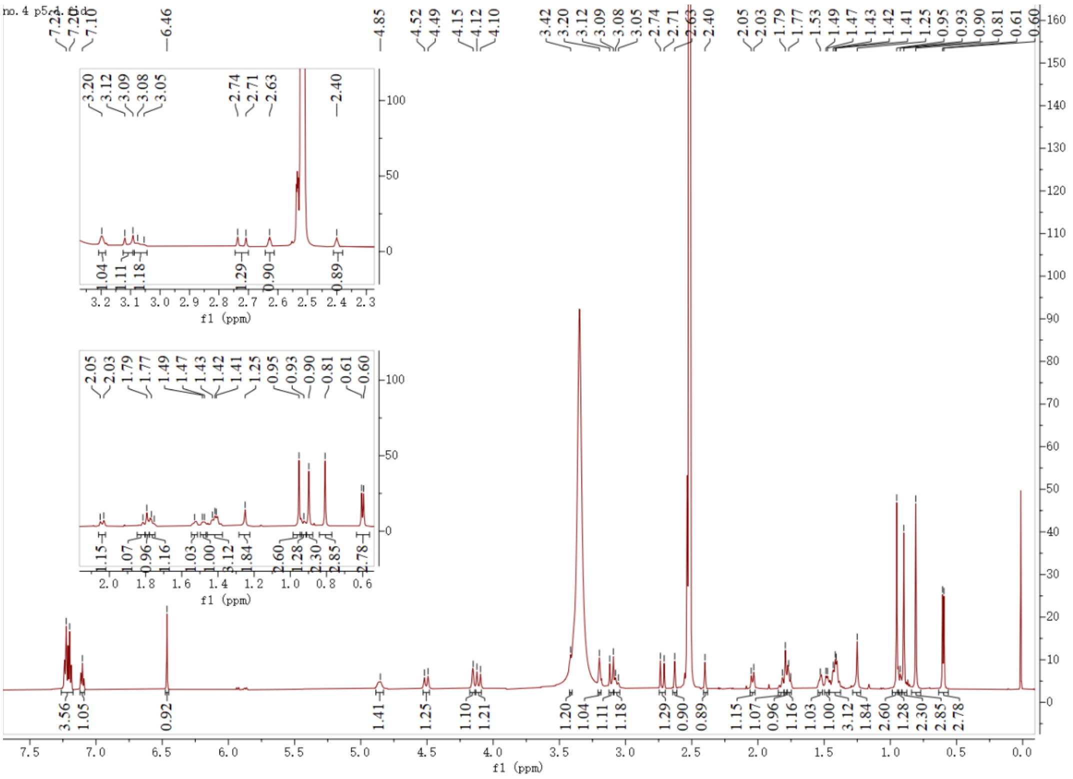

Figure S9. ^{1}H NMR spectrum of compound **2** (in DMSO-d_6; δ, ppm; *J*, Hz).

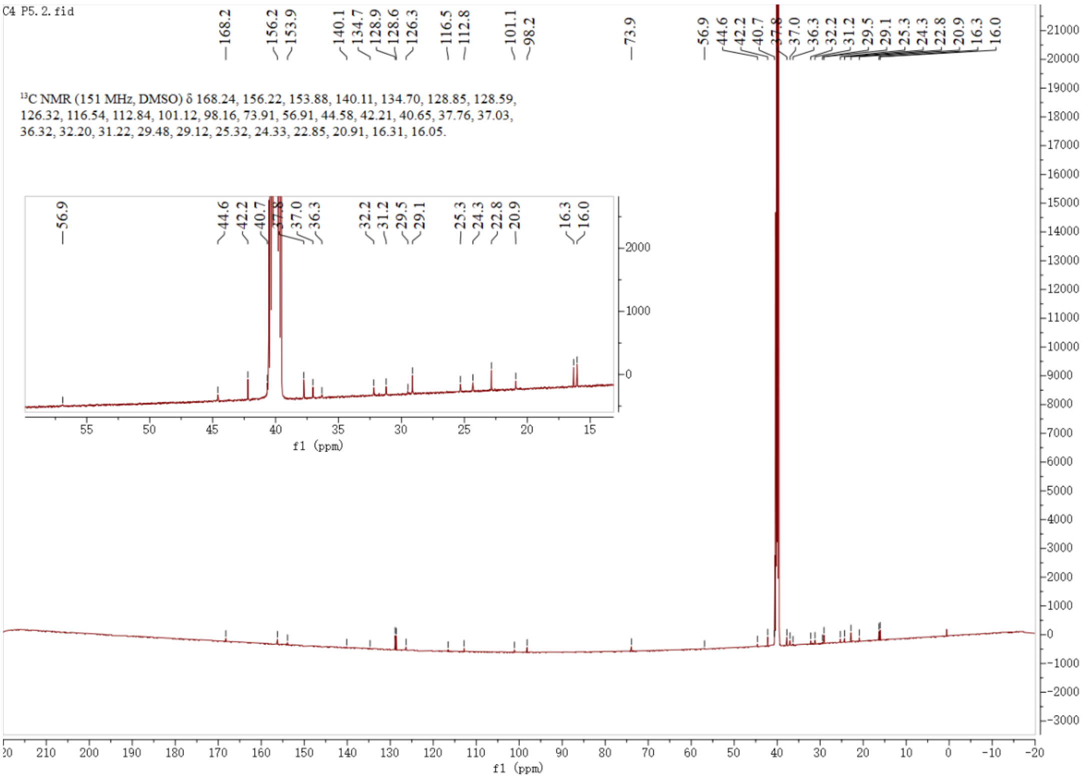

Figure S10. ^{13}C NMR spectrum of compound **2** (in DMSO-d_6; δ, ppm).

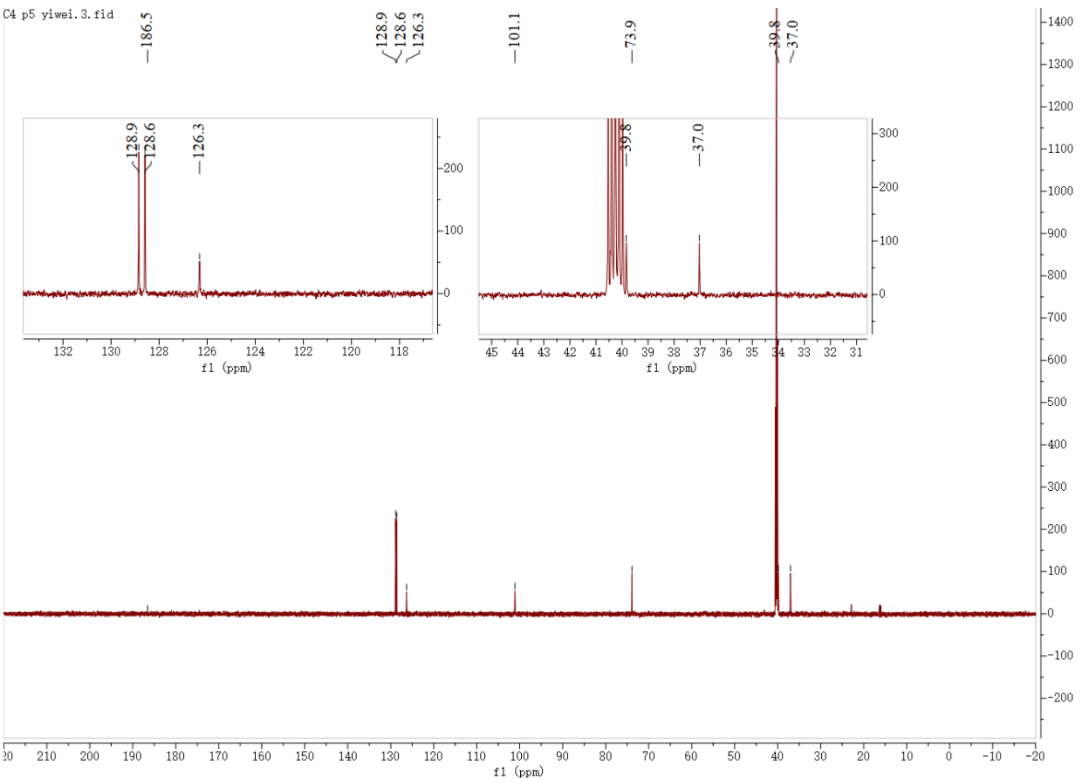

Figure S11. DEPT–90 spectrum of compound **2** (in DMSO-d_6; δ, ppm).

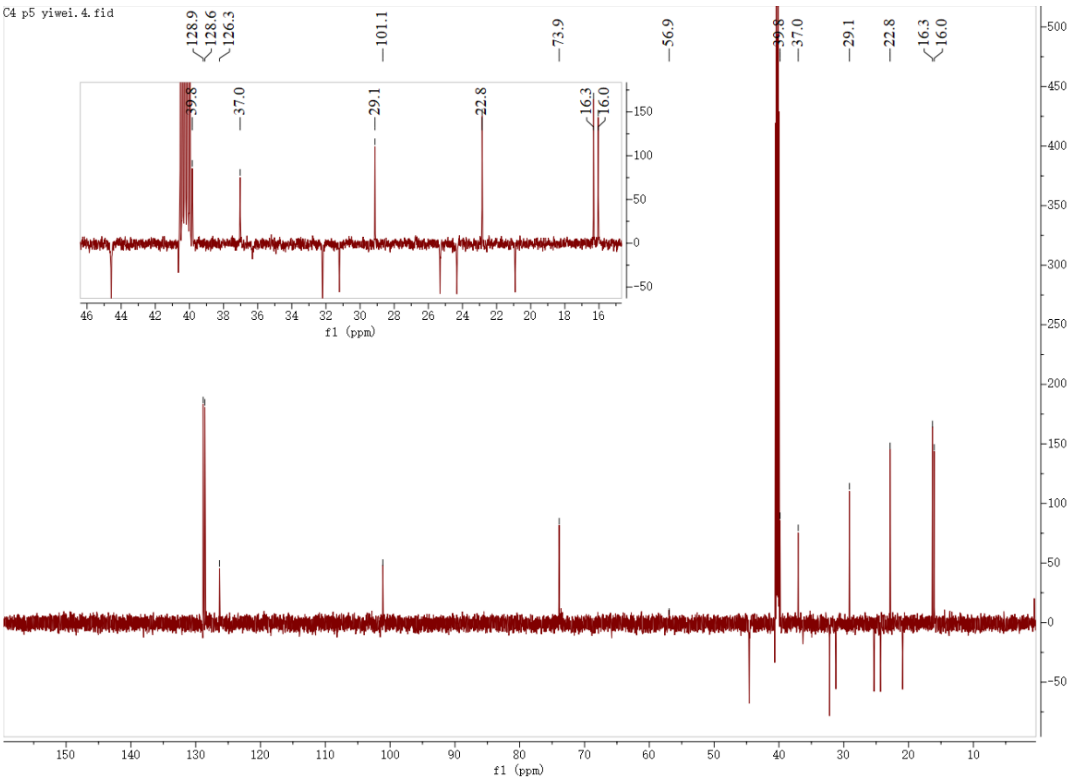

Figure S12. DEPT–115 spectrum of compound **2** (in DMSO-d_6; δ, ppm).

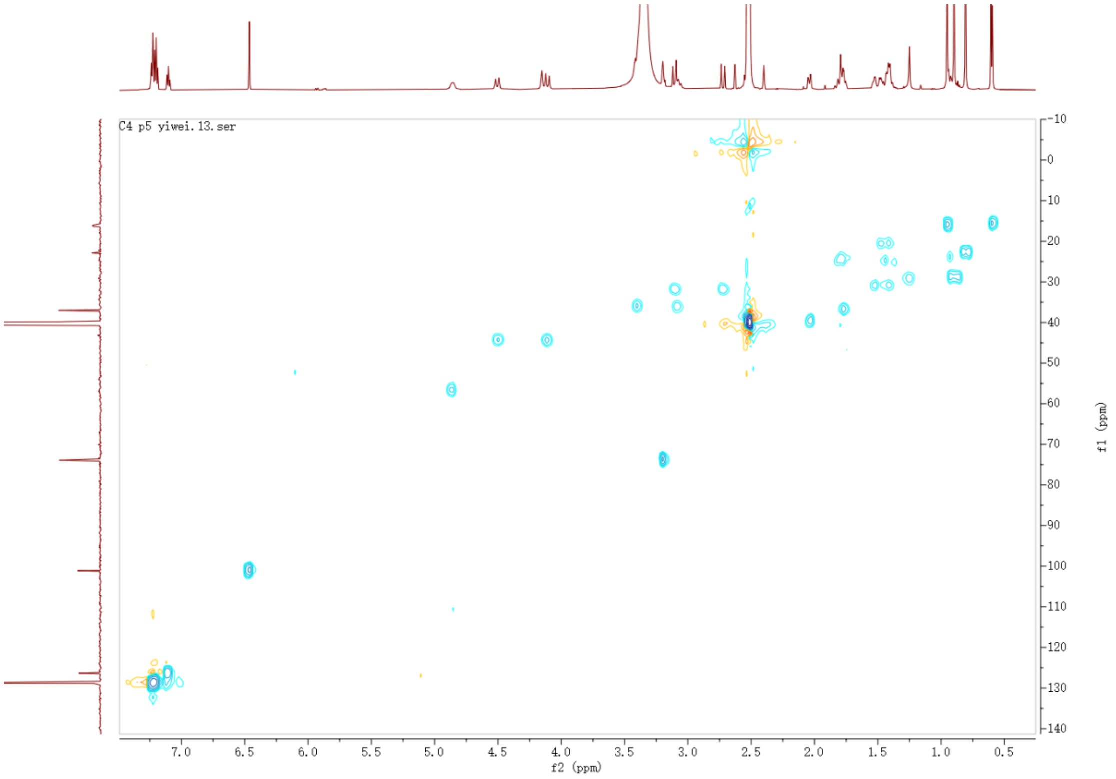

Figure S13. HMQC spectrum of compound **2** (in DMSO-*d*6; δ, ppm).

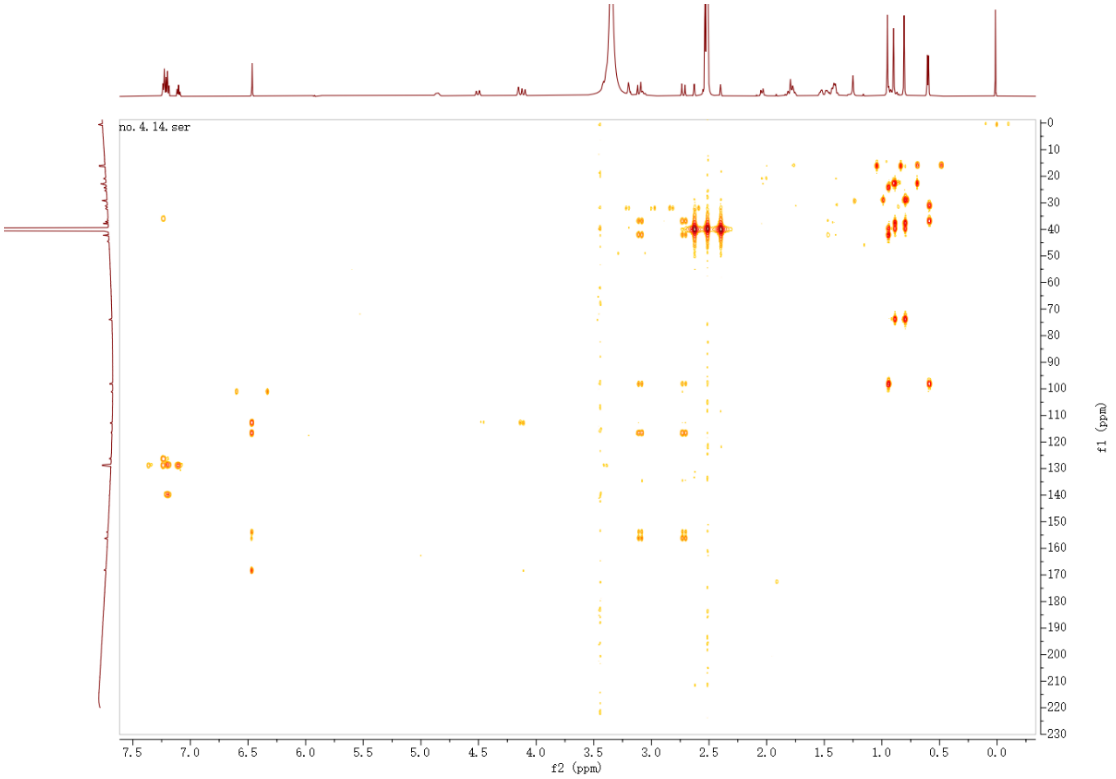

Figure S14. HMBC spectrum of compound **2** (in DMSO-*d*6; δ, ppm).

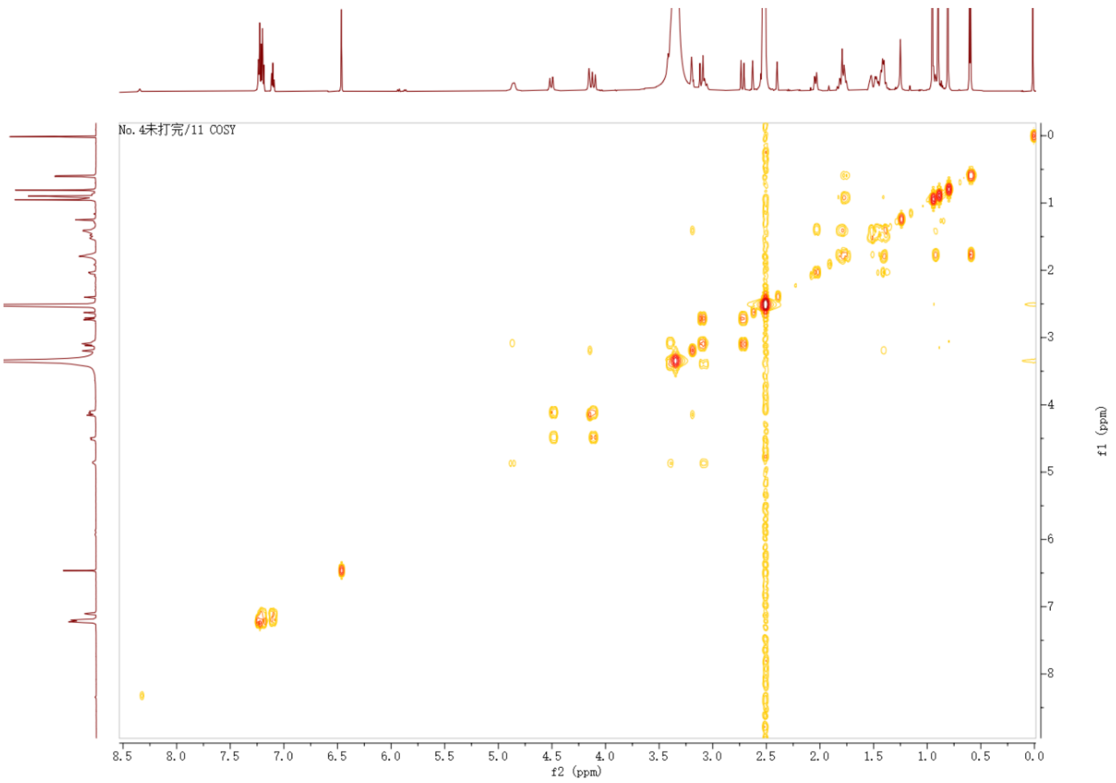

Figure S15. ^1H–^1H COSY spectrum of compound **2** (in DMSO-d_6; δ, ppm).

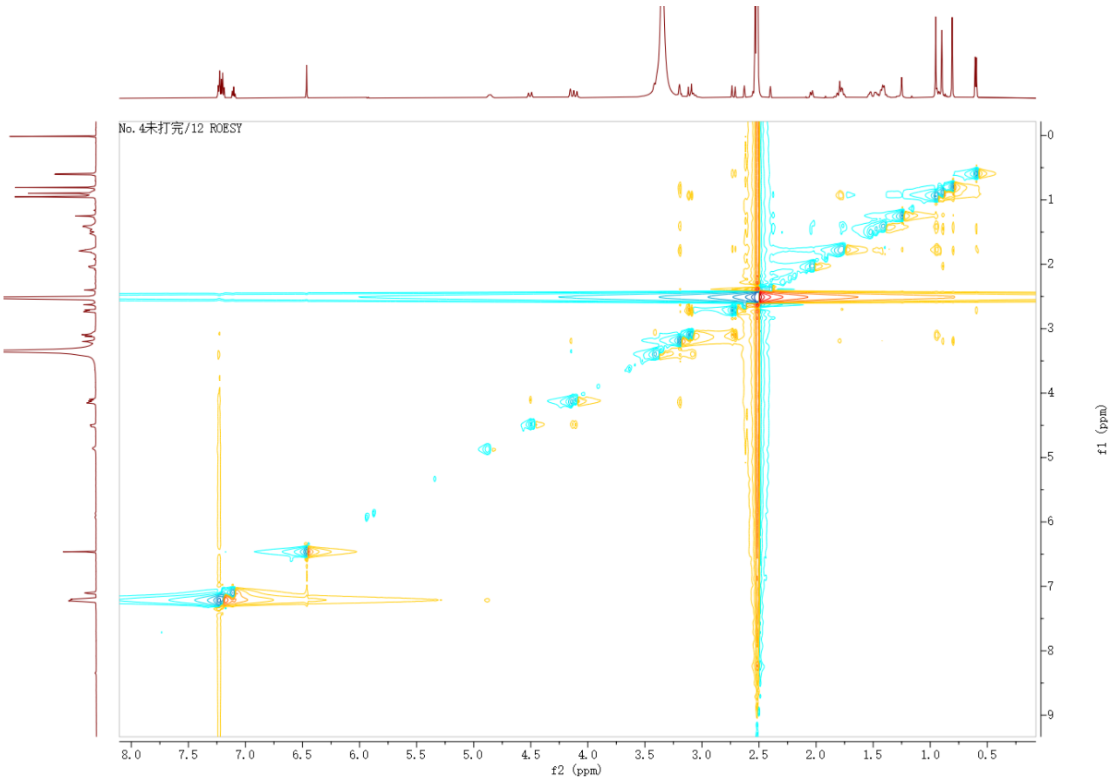

Figure S16. ROESY spectrum of compound **2** (in DMSO-d_6; δ, ppm).

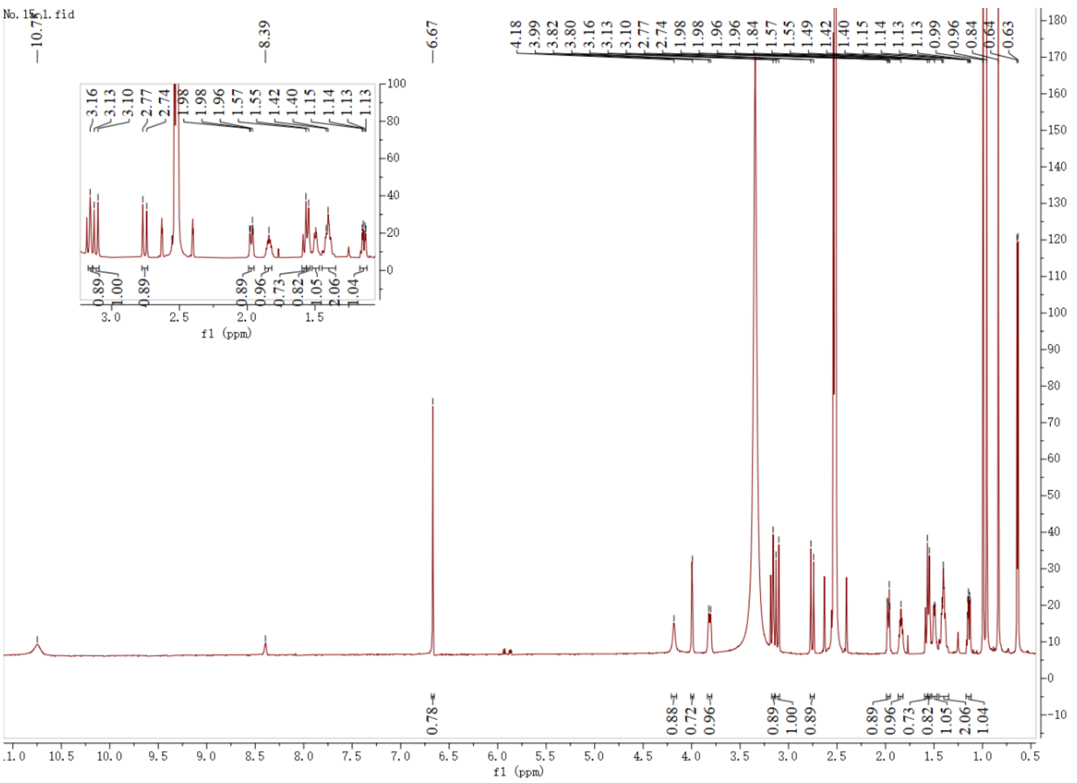

Figure S17. ^{1}H NMR spectrum of compound **3** (in DMSO-d_6; δ, ppm; *J*, Hz).

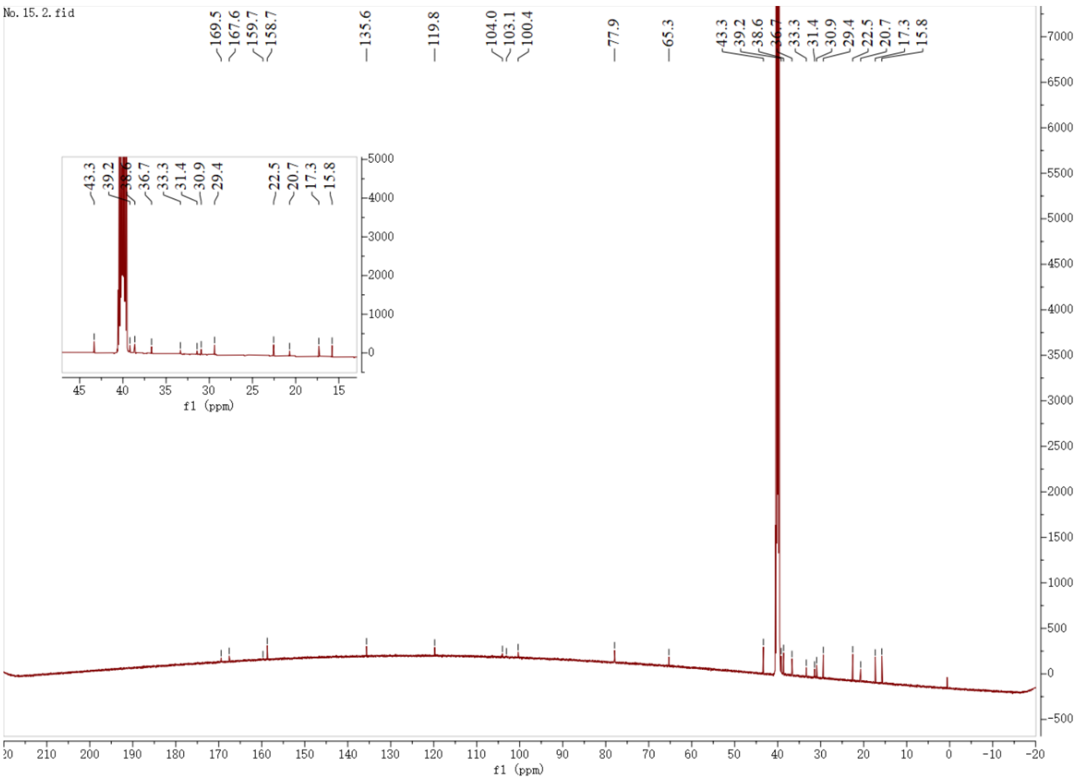

Figure S18. ^{13}C NMR spectrum of compound **3** (in DMSO-d_6; δ, ppm).

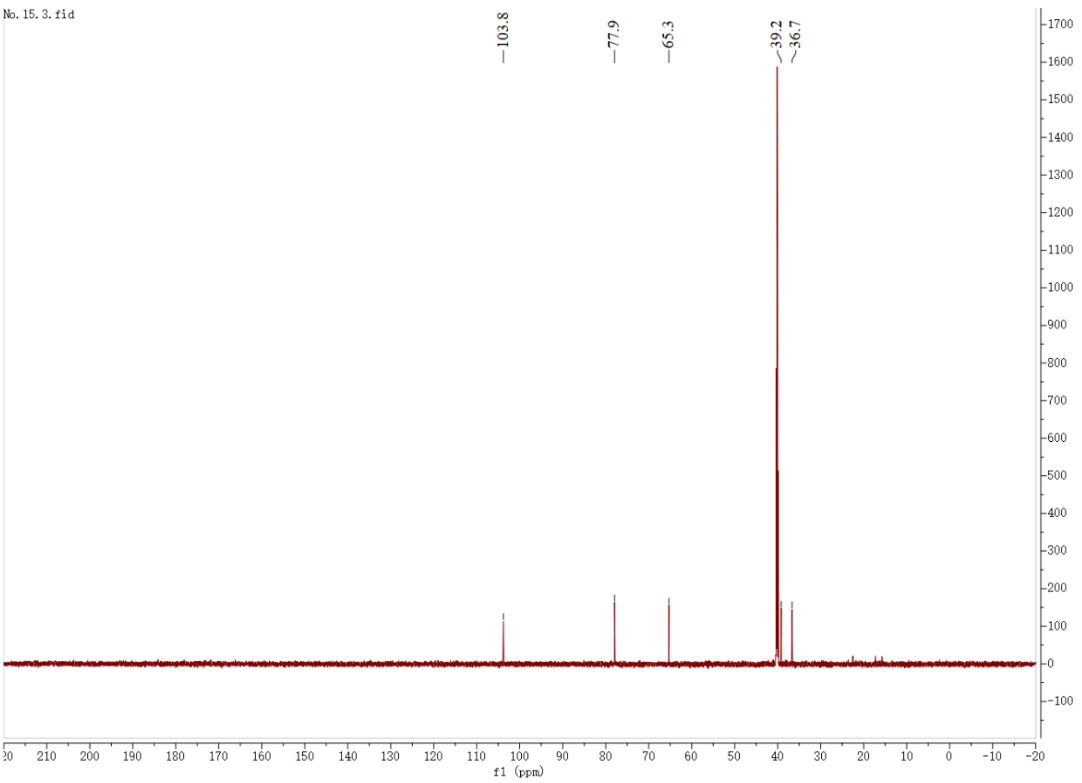

Figure S19. DEPT–90 spectrum of compound **3** (in DMSO-d_6; δ, ppm).

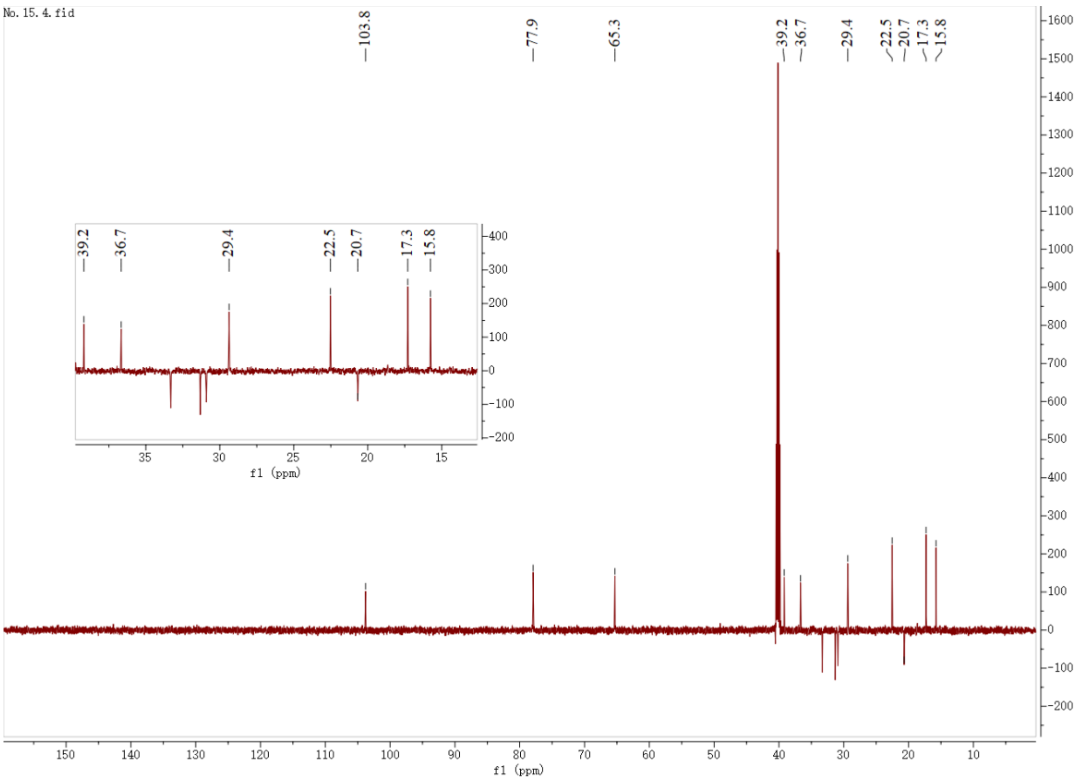

Figure S20. DEPT–115 spectrum of compound **3** (in DMSO-d_6; δ, ppm).

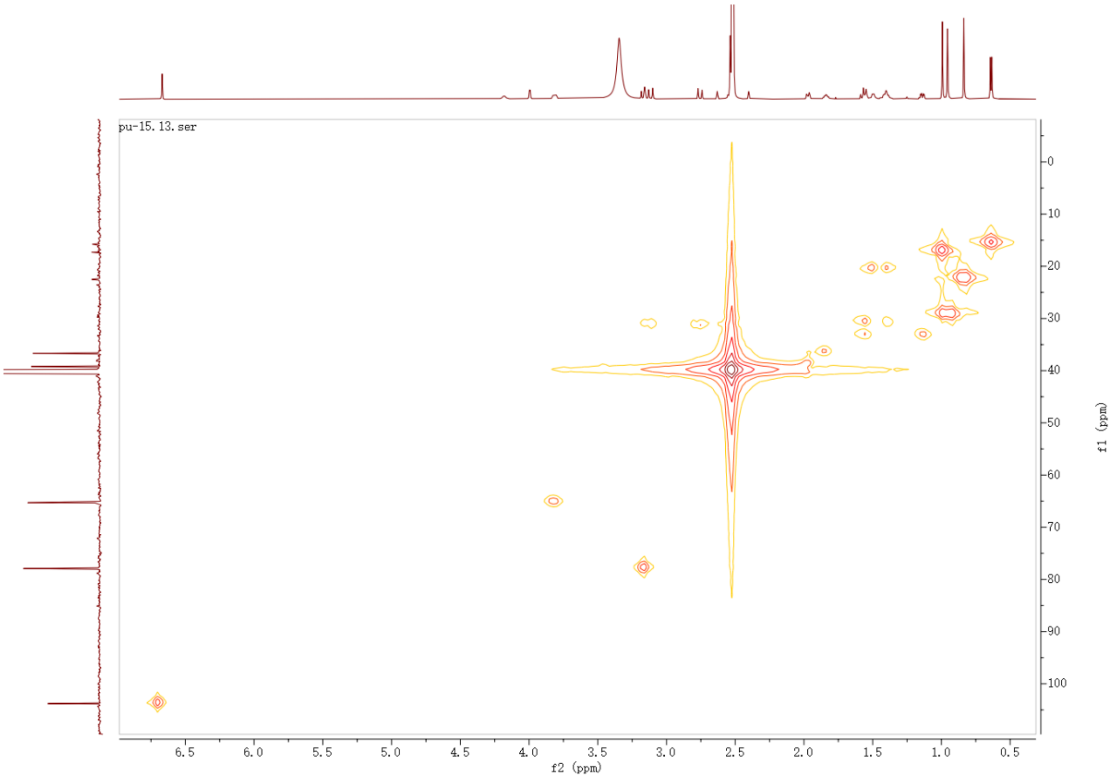

Figure S21. HMQC spectrum of compound **3** (in DMSO-*d*6; δ, ppm).

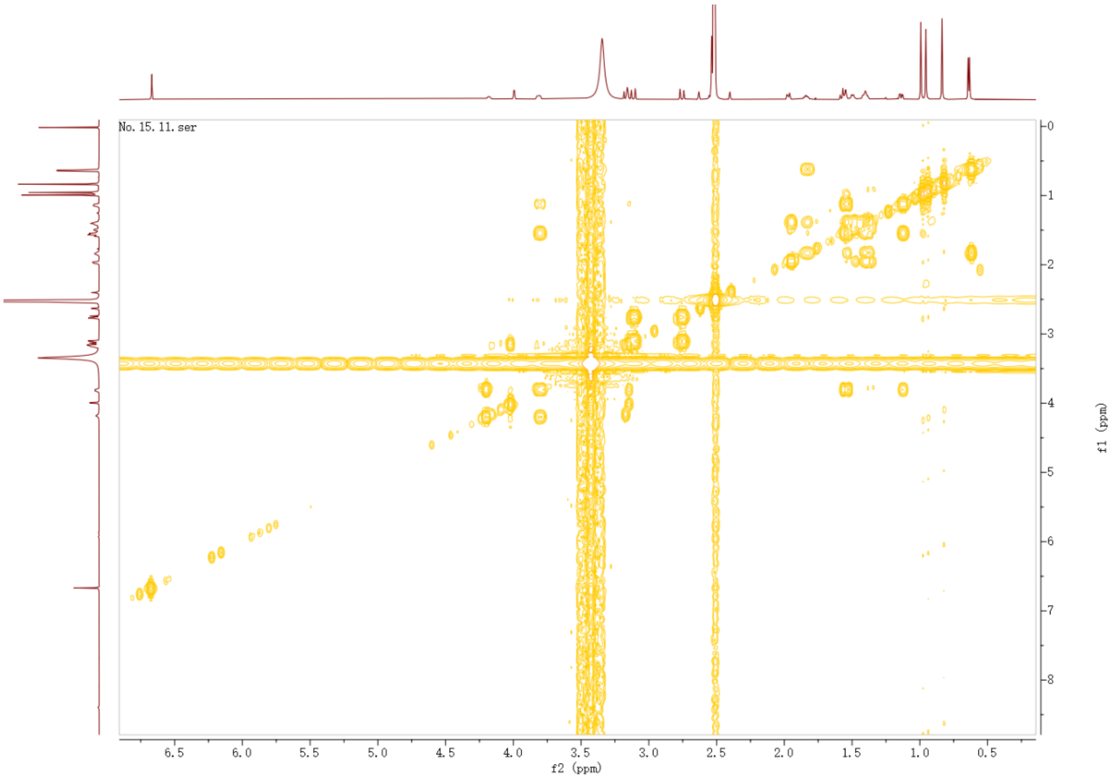

Figure S22. HMBC spectrum of compound **3** (in DMSO-*d*6; δ, ppm).

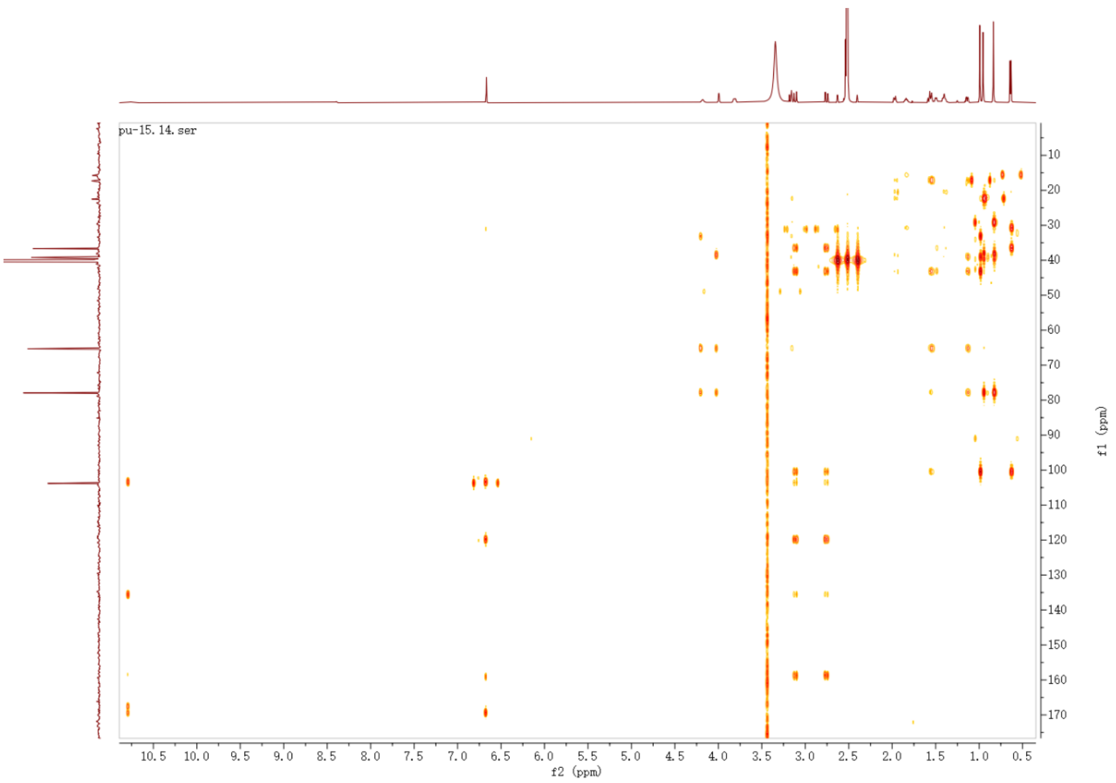

Figure S23. ^1H–^1H COSY spectrum of compound **3** (in DMSO-*d*6; δ, ppm).

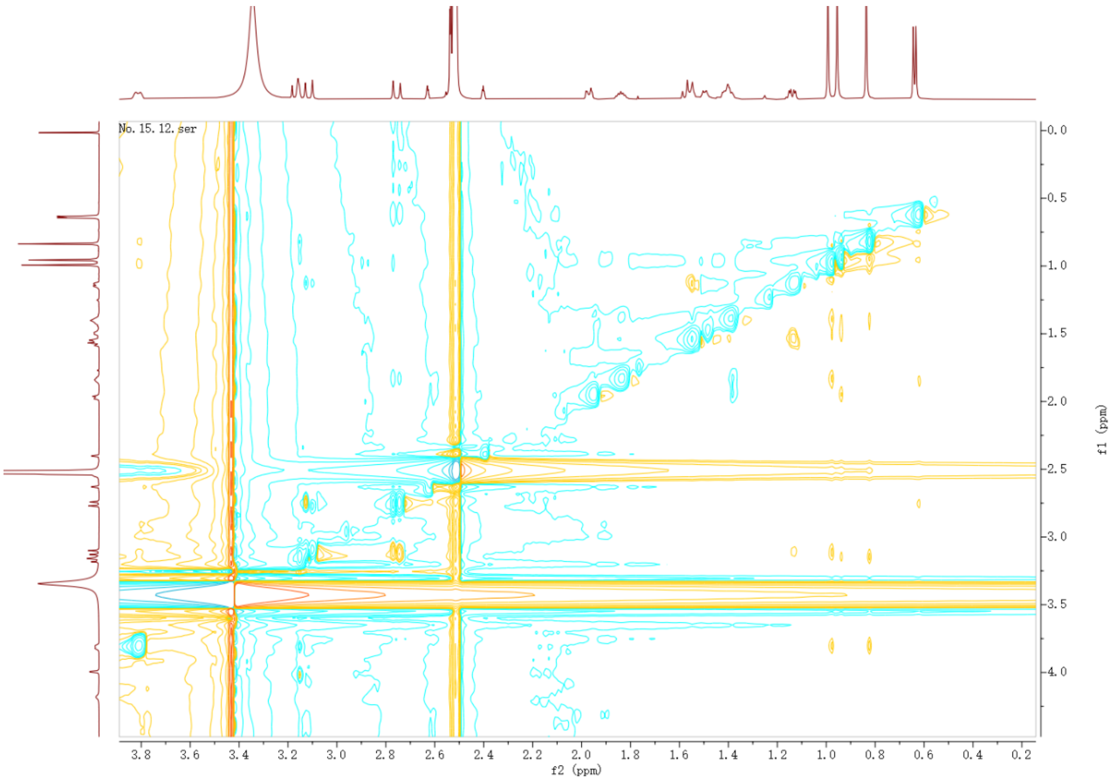

Figure S24. ROESY spectrum of compound **3** (in DMSO-*d*6; δ, ppm).

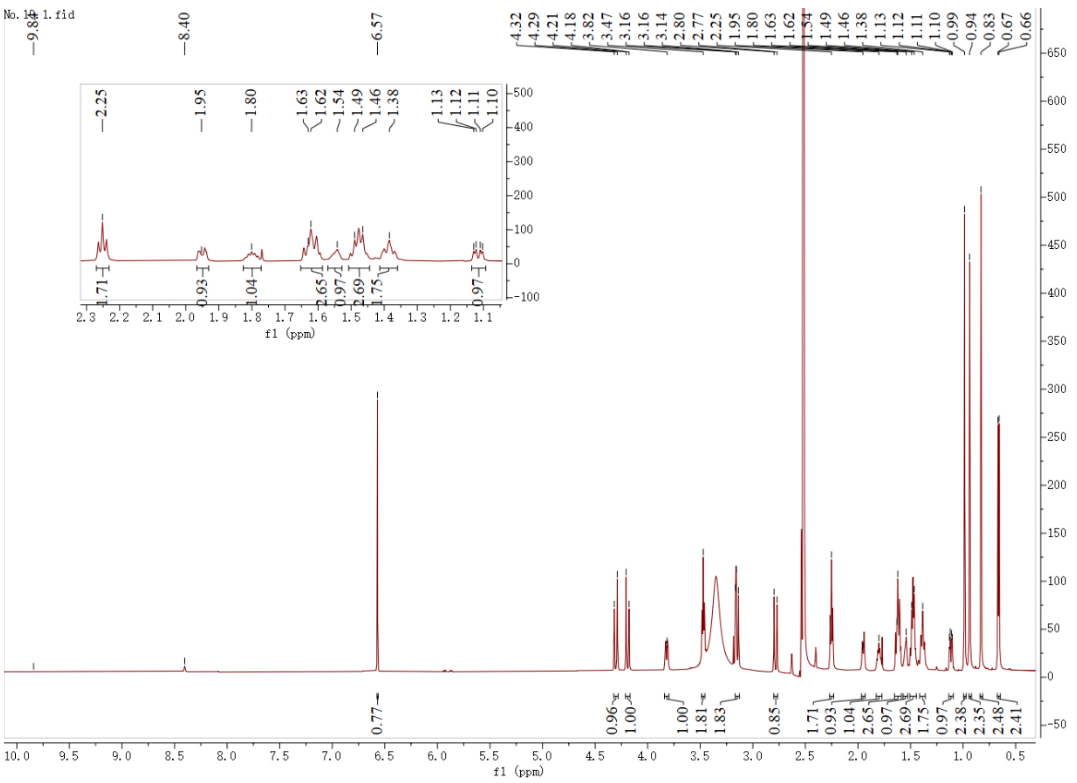

Figure S25. ^{1}H NMR spectrum of compound **4** (in DMSO-d_6; δ, ppm; *J*, Hz).

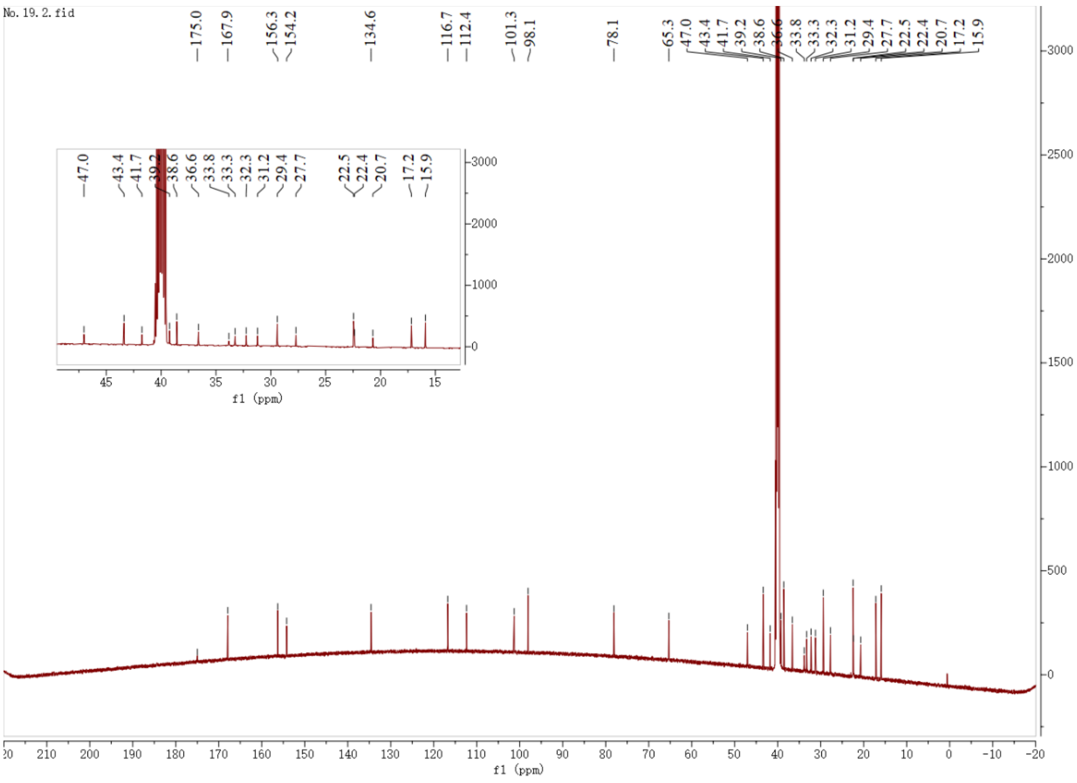

Figure S26. ^{13}C NMR spectrum of compound **4** (in DMSO-d_6; δ, ppm).

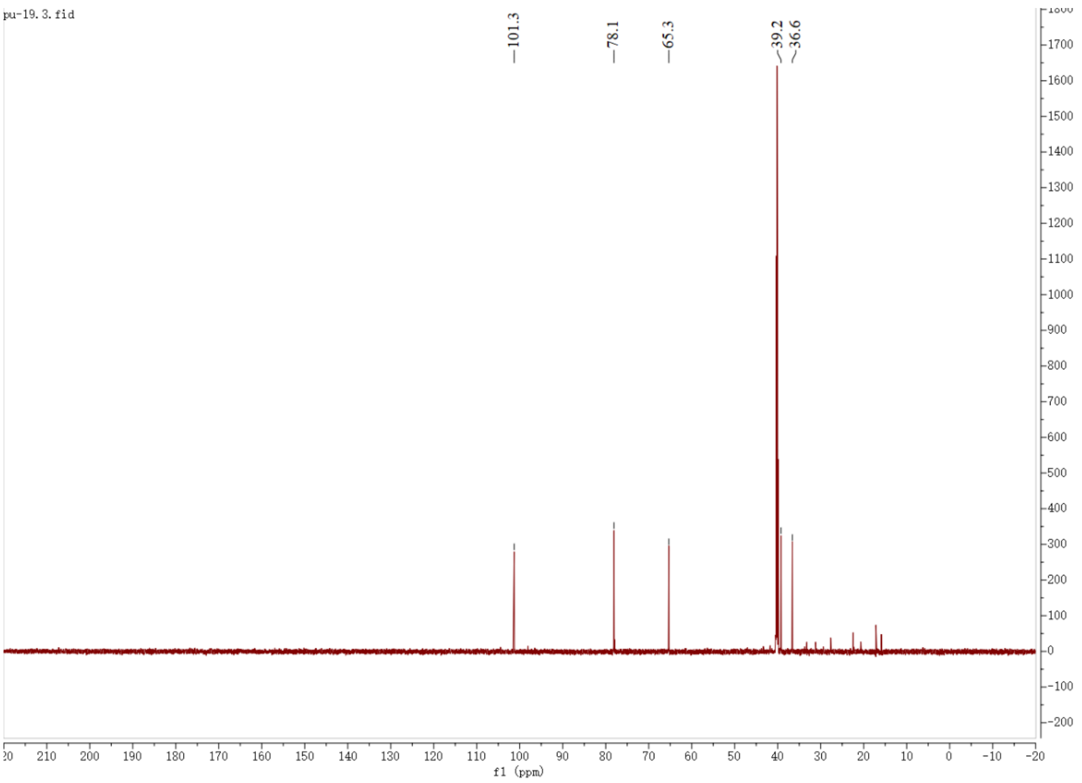

Figure S27. DEPT–90 spectrum of compound **4** (in DMSO-d_6; δ, ppm).

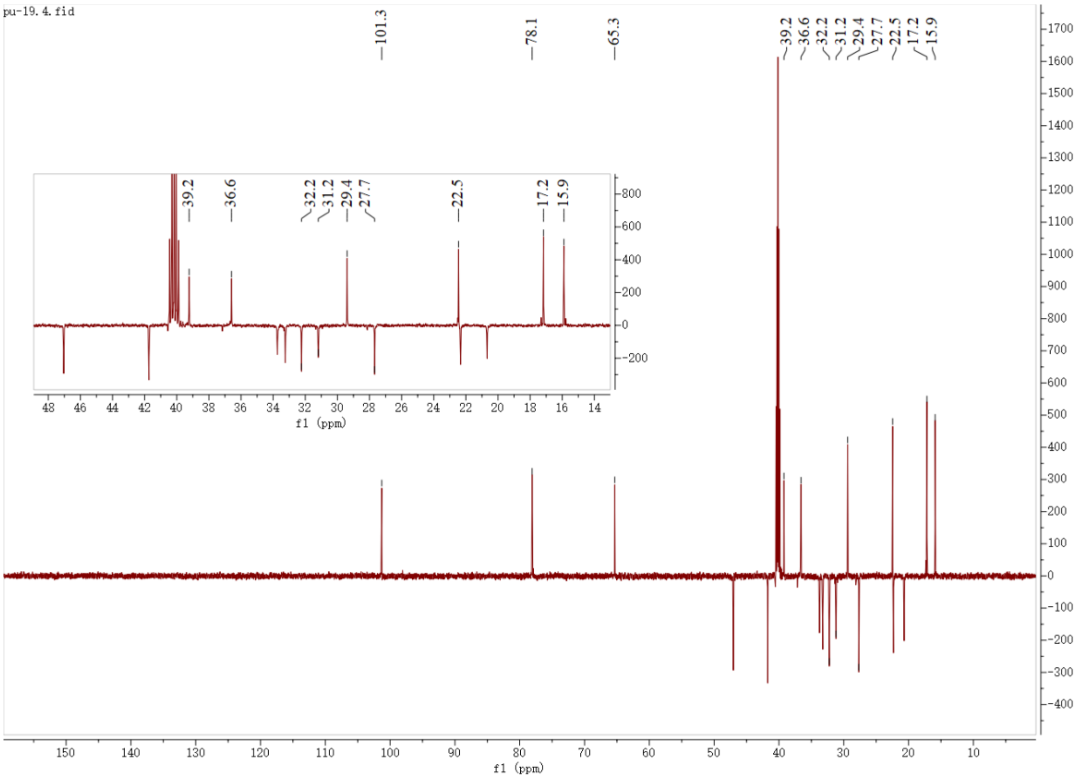

Figure S28. DEPT–115 spectrum of compound **4** (in DMSO-d_6; δ, ppm).

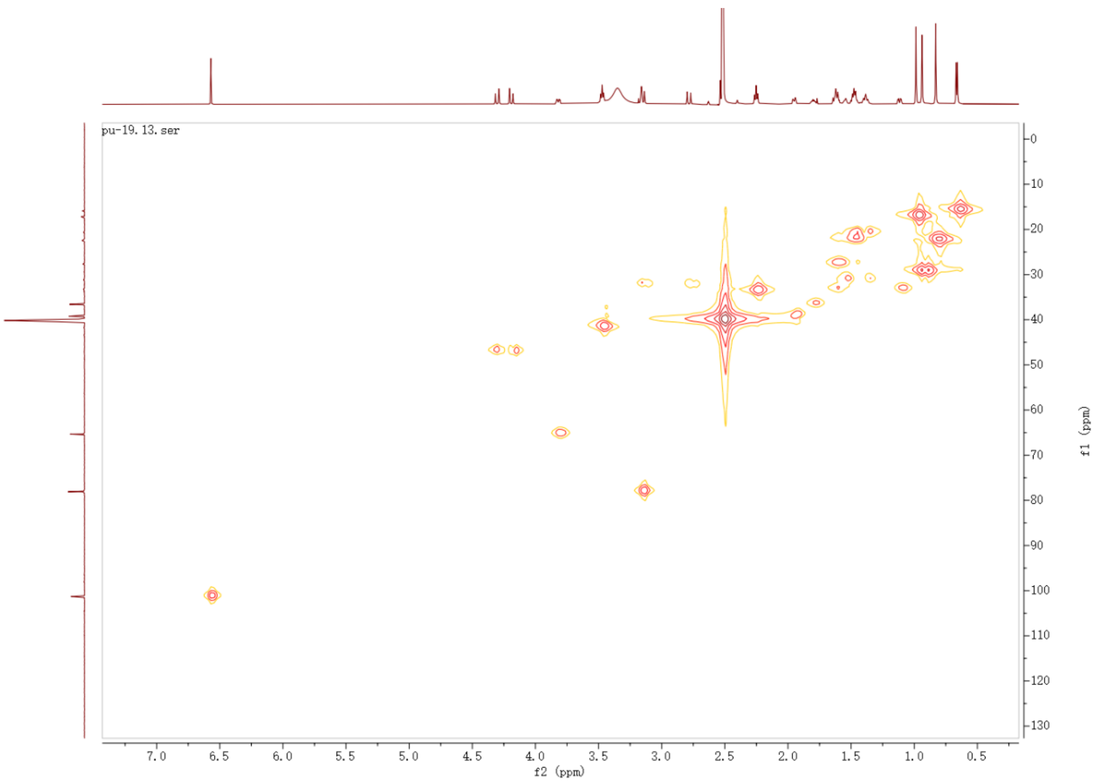

Figure S29. HMQC spectrum of compound **4** (in DMSO-*d*6; δ, ppm).

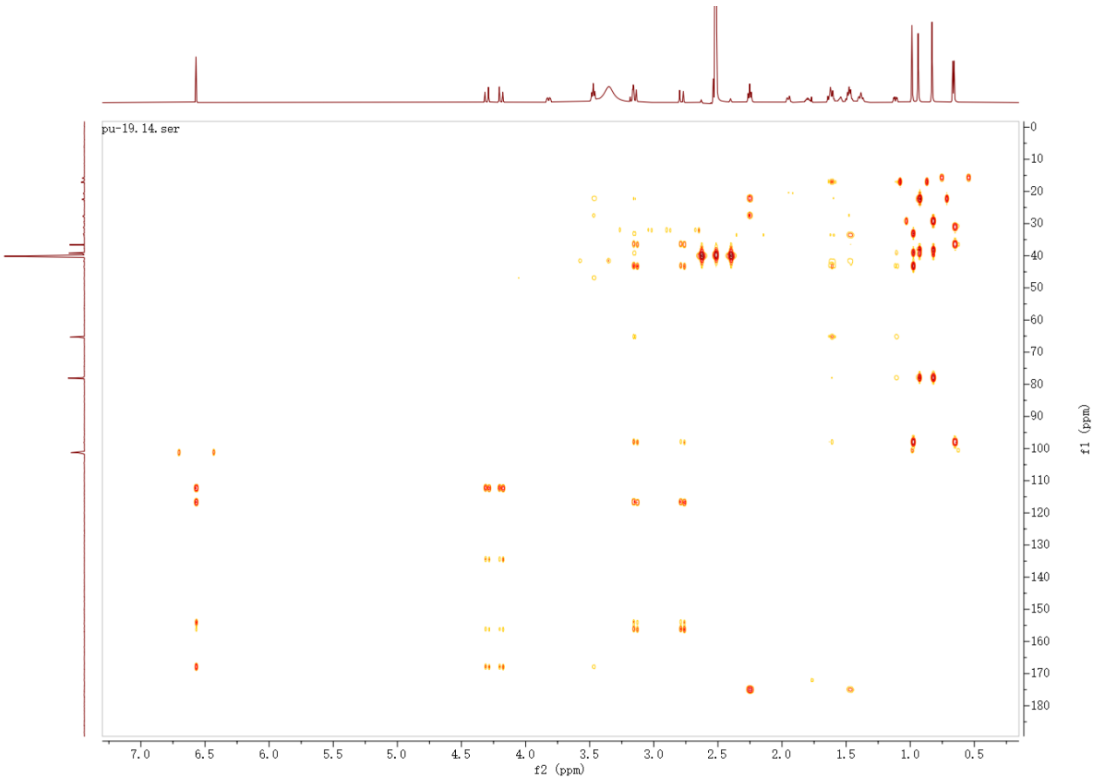

Figure S30. HMBC spectrum of compound **4** (in DMSO-*d*6; δ, ppm).

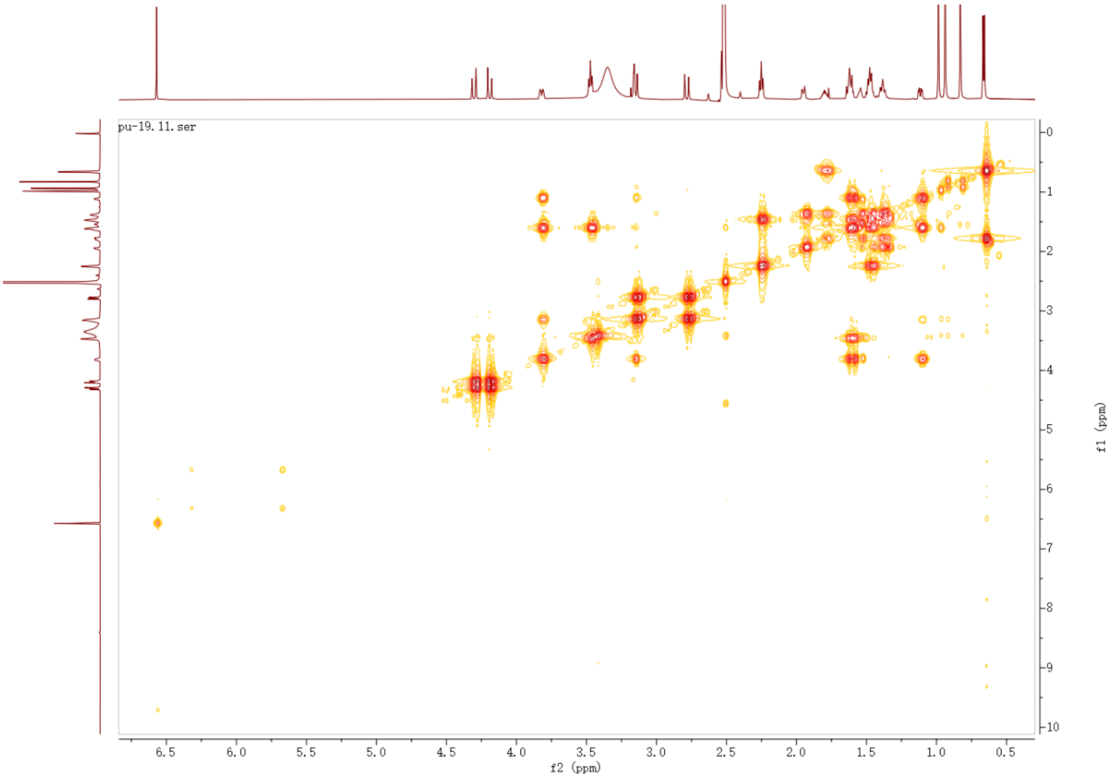

Figure S31. ^1H–^1H COSY spectrum of compound **4** (in DMSO-d_6; δ, ppm).

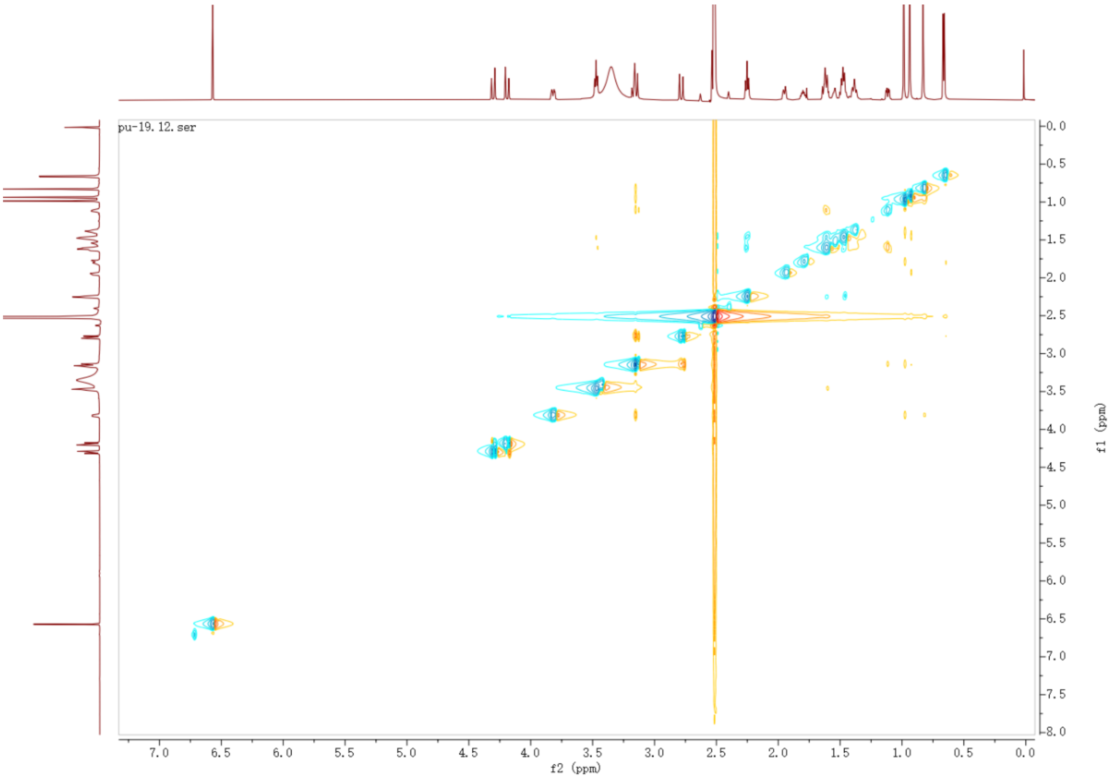

Figure S32. ROESY spectrum of compound **4** (in DMSO-d_6; δ, ppm).

Table S1. Coordinates (Ångstroms) for conformer Chartarlactam U (2*R*, 3*S*, 5*S*, 8*R*, 9*R*, 10*S*) at the lowest energy (Energy: -1710.416026 Hartree, Solvent: CH_3OH, Boltzmann %: 48.40).

C	3.58768900	-16.67023700	-1.36049800
C	4.49954900	-16.59742800	-2.57780500
C	4.77180100	-15.14734200	-3.01097000
C	3.41420800	-14.42845400	-3.22651500
C	2.38144200	-14.53660700	-2.06858300
C	2.25321700	-16.00705900	-1.63974900
C	3.53780600	-12.99119200	-3.71193200
C	2.19840200	-12.46193400	-4.18958000
C	1.07937500	-12.60949100	-3.16639400
C	1.00707400	-14.04324100	-2.62267900
C	-0.16099500	-14.21684700	-1.61165300
H	2.95793900	-14.98790800	-4.04278700
O	0.66086000	-14.90743000	-3.76659700
C	-0.55993400	-15.44519000	-3.55524900
C	-1.12862300	-15.07872200	-2.34607500
C	2.73193100	-13.68739700	-0.83851800
O	3.42903500	-18.01170200	-0.89675000
O	3.92136600	-17.28993000	-3.68457400
C	5.54132600	-15.19241800	-4.33791000
C	5.69803700	-14.47696200	-1.99398200
C	-1.21093500	-16.30349000	-4.41081300
C	-2.46259800	-16.76530600	-4.02419500
C	-3.07097900	-16.41551100	-2.83368400
C	-2.38160100	-15.55766400	-1.97956100
O	-2.96988900	-15.22396700	-0.79184600
C	-0.81664800	-16.91196300	-5.70944900
N	-1.96872100	-17.73734900	-6.02478100
C	-2.94280300	-17.71040400	-5.05476400
O	-3.97048700	-18.37676300	-5.06454800
C	-1.95509500	-18.72941000	-7.07315000
C	-1.69230500	-20.15126500	-6.55095700
C	-0.48021500	-20.22356800	-5.67666900
C	-0.58181400	-19.98340200	-4.30801700
C	0.53887100	-19.93840600	-3.49760100
C	1.79031100	-20.15089200	-4.05282600
C	1.91965000	-20.40604600	-5.40933100
C	0.78719600	-20.43935800	-6.20771800
O	2.88439700	-20.08131800	-3.20844900
C	-0.23463400	-12.12599300	-3.76657900
H	4.09258900	-16.17650200	-0.53234200
H	5.45649500	-17.06459300	-2.31251400
H	1.64065400	-16.10022300	-0.74371100

H	1.76092000	-16.56800600	-2.43267700
H	3.93514000	-12.34309000	-2.93000000
H	4.24768600	-12.93357800	-4.53489300
H	1.90752000	-12.99917800	-5.09436500
H	2.28423700	-11.41058900	-4.47026500
H	1.31018800	-11.97448400	-2.30639100
H	-0.57194100	-13.24690100	-1.32827400
H	0.16981700	-14.69321800	-0.68910700
H	3.11406900	-12.70427300	-1.09136400
H	1.86045300	-13.54110800	-0.20407500
H	3.47776300	-14.17349200	-0.22090400
H	2.89779900	-18.50537200	-1.53440400
H	3.73414800	-18.19850100	-3.41224700
H	5.95514900	-14.21849300	-4.59043900
H	6.37699000	-15.88712800	-4.25792300
H	4.91379300	-15.52692000	-5.15848900
H	5.87641200	-13.43839600	-2.26410000
H	5.32582400	-14.48756900	-0.97584500
H	6.66604200	-14.97777100	-1.98915000
H	-4.04198300	-16.80416700	-2.56612400
H	-2.38311200	-14.63346700	-0.30604200
H	-0.63949300	-16.16702800	-6.49136200
H	0.08994000	-17.51454700	-5.61956700
H	-2.91651500	-18.71338900	-7.58560100
H	-1.18840900	-18.44081900	-7.79036700
H	-2.57269800	-20.47222500	-5.99920300
H	-1.58861100	-20.81341600	-7.41156600
H	-1.55672200	-19.82358900	-3.86875900
H	0.44850200	-19.75734600	-2.43571000
H	2.89950000	-20.56259800	-5.84425100
H	0.89755700	-20.63001100	-7.26806500
H	3.63043000	-20.53351200	-3.61954800
H	-0.12525400	-11.10099400	-4.11799000
H	-0.52128100	-12.73799800	-4.61928600
H	-1.06056800	-12.13791800	-3.05915700

Table S2. Coordinates (Ångstroms) for conformer Chartarlactam V (3R, 5S, 8R, 9R, 10S) at the lowest energy (Energy: -1713.727423 Hartree, Solvent: CH3OH, Boltzmann %: 50.78).

C	-12.14395500	-1.44445000	-0.92740700
C	-11.79627700	-1.43165000	-2.40415500
C	-10.68109500	-2.43061000	-2.75411400
C	-9.46919300	-2.19234200	-1.81274700
C	-9.77441800	-2.12875300	-0.28750900
C	-10.93603100	-1.15131900	-0.05582400

C	-8.28296100	-3.10277600	-2.09721600
C	-7.04485900	-2.63928600	-1.35370300
C	-7.26172600	-2.46134600	0.14401900
C	-8.49636900	-1.59352400	0.42881700
C	-8.69036200	-1.35777800	1.94934500
H	-9.14529900	-1.18159500	-2.06141800
O	-8.19912200	-0.25512100	-0.13203200
C	-8.00940400	0.60412200	0.89008400
C	-8.25778200	0.05131800	2.13526500
C	-10.12485000	-3.48484700	0.33846900
O	-11.36250700	-0.12551200	-2.80838500
C	-10.25659700	-2.17844400	-4.20729700
C	-11.25410800	-3.84757600	-2.70430000
C	-7.58297000	1.90854100	0.76668800
C	-7.40365400	2.63633400	1.93421400
C	-7.63750400	2.12061200	3.19644200
C	-8.08063800	0.80223900	3.29013900
O	-8.34419900	0.18582300	4.48079300
C	-7.19580400	2.73209600	-0.41067700
N	-6.82336700	3.99449600	0.20241700
C	-6.85843400	3.95662600	1.57089700
O	-6.46749500	4.84958600	2.32433800
C	-5.98700500	-1.93058700	0.78833900
C	-6.13960800	5.04097900	-0.52521400
C	-4.69047200	4.68976900	-0.83779300
C	-3.88261100	4.33005500	0.40178200
C	-2.46109800	3.89766300	0.05223500
C	-2.43488100	2.65331400	-0.78321500
C	-2.06450200	2.68512100	-2.12396400
C	-2.09485500	1.53776500	-2.90381200
C	-2.50107500	0.33220900	-2.35251300
C	-2.86897300	0.28316500	-1.01499300
C	-2.83402900	1.43198400	-0.24109300
O	-3.80135500	5.41824400	1.30912700
H	-12.92229300	-0.70014200	-0.73488100
H	-12.59536900	-2.40268400	-0.67113700
H	-12.68404300	-1.70908400	-2.98810100
H	-11.24428200	-1.16835400	0.98948800
H	-10.59258600	-0.14147300	-0.26728300
H	-8.50951700	-4.13588600	-1.83102700
H	-8.06756100	-3.11101700	-3.16381700
H	-6.22388800	-3.34132200	-1.51224300
H	-6.71579800	-1.68443300	-1.76835200
H	-7.47990400	-3.43960900	0.58082000

H	-9.72382200	-1.49061700	2.26327600
H	-8.10386700	-2.05848700	2.54092900
H	-9.51003300	-4.29840000	-0.03222800
H	-10.00746600	-3.45760100	1.41961800
H	-11.15764300	-3.75605300	0.15050900
H	-12.04224400	0.50194300	-2.53958900
H	-9.65968200	-3.00161700	-4.59348100
H	-11.13584600	-2.09039300	-4.84499500
H	-9.68549500	-1.26032700	-4.30844300
H	-10.48355800	-4.58392200	-2.92161700
H	-11.69588100	-4.11103600	-1.74998700
H	-12.02824400	-3.96166100	-3.46339700
H	-7.46384900	2.73024600	4.07380400
H	-8.17524900	0.80866200	5.19702900
H	-6.35539500	2.29239500	-0.95520500
H	-8.01180600	2.86615400	-1.12589300
H	-6.06039900	-1.83258100	1.86867800
H	-5.15969600	-2.60760800	0.57856900
H	-5.72618800	-0.95566600	0.38222800
H	-6.68906700	5.24720200	-1.44430300
H	-6.18704200	5.93561100	0.09109000
H	-4.65393700	3.86314600	-1.54800600
H	-4.22364300	5.54781300	-1.32532900
H	-4.36394500	3.47769600	0.89549300
H	-1.95638500	4.71662800	-0.46065800
H	-1.93170600	3.73816400	0.99167900
H	-1.75016300	3.62390200	-2.56296700
H	-1.79965600	1.58644400	-3.94381200
H	-2.52694800	-0.56408400	-2.95812000
H	-3.17898800	-0.65402600	-0.57229700
H	-3.11777500	1.38107000	0.80300500
H	-4.64896200	5.45303500	1.78592400

Table S3. Coordinates (Ångstroms) for conformer Chartarlactam W (2R, 3S, 5S, 8R, 9R, 10S) at the lowest energy (Energy: -1399.716015 Hartree, Solvent: CH3OH, Boltzmann %: 52.75).

C	-11.82684100	-2.87201500	0.03447700
C	-10.92063100	-3.40938700	-1.05877200
C	-9.42314400	-3.23283200	-0.74057800
C	-9.14777800	-1.75081100	-0.37851300
C	-10.08707800	-1.11281000	0.68383800
C	-11.54405400	-1.40147300	0.29871600
C	-7.68847400	-1.44862300	-0.06826900
C	-7.43904700	0.04711800	-0.01604600
C	-8.37553800	0.79425600	0.92657400

C	-9.84091700	0.42836300	0.65502300
C	-10.81520500	1.22408400	1.56530700
H	-9.38350500	-1.21392100	-1.29800400
O	-10.13234500	0.89433800	-0.71714300
C	-11.01360800	1.89885300	-0.66443000
C	-11.46111700	2.17891300	0.62349400
C	-9.83666900	-1.60204600	2.11660400
O	-13.19468600	-3.08236700	-0.28958400
O	-11.32739900	-2.71240700	-2.24548600
C	-9.02102800	-4.24422800	0.33446000
C	-8.63266800	-3.59694800	-2.00410600
C	-11.48169200	2.63072900	-1.73777800
C	-12.40568000	3.64750300	-1.47614000
C	-12.86868400	3.95082200	-0.22130400
C	-12.38097200	3.18975600	0.85118300
O	-12.84601100	3.48967200	2.09317100
C	-11.20269300	2.59009600	-3.17729300
N	-12.00476100	3.60909000	-3.71937700
C	-12.75510300	4.29280100	-2.76962600
O	-13.51723500	5.21145800	-2.98713000
C	-8.10043900	2.29064700	0.84212300
O	-10.46252700	1.87453600	-3.81981000
H	-11.66225000	-3.45716300	0.93685200
H	-11.12705500	-4.47867000	-1.18110100
H	-12.23083100	-1.07756300	1.07971500
H	-11.79645600	-0.84786600	-0.60299600
H	-7.38409600	-1.91030400	0.87167900
H	-7.04647200	-1.88120100	-0.83331600
H	-7.55805000	0.46401500	-1.01753400
H	-6.40784300	0.24766400	0.27988100
H	-8.17299500	0.46870800	1.95041400
H	-11.55180000	0.57248600	2.03351900
H	-10.27247500	1.71545200	2.37344200
H	-10.31030400	-0.94442200	2.84285400
H	-10.25786600	-2.58631200	2.28356600
H	-8.78402500	-1.65792700	2.37294600
H	-13.29524100	-2.78482800	-1.20423800
H	-10.99925500	-3.19250300	-3.01110900
H	-9.60132700	-4.16958700	1.24685800
H	-7.97348000	-4.12897600	0.60374000
H	-9.14525100	-5.25902100	-0.04361700
H	-8.72469300	-2.83571500	-2.77656900
H	-7.57402400	-3.72286000	-1.79136800
H	-8.98250800	-4.54785900	-2.41087200

H	-13.58322400	4.74248400	-0.05434300
H	-12.44894000	2.88997400	2.73584900
H	-12.02669700	3.82370300	-4.70210900
H	-8.70218200	2.87990100	1.53067300
H	-7.05569600	2.48624900	1.07796300
H	-8.28486300	2.66199900	-0.16351600

Table S4. Coordinates (Ångstroms) for conformer Chartarlactam X (2R, 3S, 5S, 8R, 9R, 10S) at the lowest energy (Energy: -1671.365832 Hartree, Solvent: CH3OH, Boltzmann %: 55.72).

C	-8.10037000	-2.39713900	-0.80541700
C	-7.62904800	-2.66634900	0.61657400
C	-8.82421200	-2.69277100	1.59063200
C	-9.58211300	-1.34590800	1.45973500
C	-9.98836000	-0.91354400	0.01983600
C	-8.77282500	-1.05188400	-0.91100500
C	-10.74731800	-1.18362000	2.42399700
C	-11.23057300	0.25497900	2.45226900
C	-11.54767100	0.83648200	1.07755100
C	-10.39560800	0.59822800	0.09431300
C	-10.65853600	1.22480900	-1.30679200
H	-8.83742800	-0.60972100	1.75729500
O	-9.23243600	1.32409500	0.62587200
C	-8.72187600	2.10482000	-0.35273600
C	-9.50825200	2.15554600	-1.49123000
C	-11.16706600	-1.70941900	-0.55611400
O	-6.99524800	-2.38723700	-1.73934900
O	-6.71119400	-1.68027500	1.04673300
C	-8.25727200	-2.82824500	3.00904800
C	-9.67833700	-3.93570200	1.33772200
C	-7.49718200	2.72441300	-0.30712200
C	-7.08832200	3.42335000	-1.43435800
C	-7.85941300	3.53775600	-2.57562400
C	-9.08890400	2.88209100	-2.60074600
O	-9.82730300	2.94765000	-3.74970100
C	-6.39443500	2.65642400	0.68739800
N	-5.32707800	3.36861900	0.01326500
C	-5.68385500	3.84768900	-1.22952700
O	-4.95243000	4.45603400	-1.99787900
C	-11.91463100	2.30811100	1.22210200
C	-3.96490200	3.33834800	0.48931000
C	-3.32284900	1.96292600	0.32825400
C	-3.11534400	1.56897700	-1.12699100
C	-2.85048700	0.07411600	-1.31753500
C	-4.07502600	-0.76566200	-1.07866100

O	-4.16930000	-1.60951500	-0.19551100
O	-5.05712200	-0.49390500	-1.93559500
H	-8.77440700	-3.19740200	-1.11541400
H	-7.15375300	-3.65962700	0.64625900
H	-9.05408700	-0.88446100	-1.94972700
H	-8.02942200	-0.30625600	-0.64841000
H	-11.57149300	-1.85138600	2.16989000
H	-10.43875600	-1.46409900	3.42910200
H	-12.11565800	0.34391000	3.08463900
H	-10.46111800	0.87700900	2.91312300
H	-12.41106000	0.30562000	0.66752400
H	-11.62977700	1.71924800	-1.34036600
H	-10.67632200	0.46228100	-2.08457400
H	-10.84390100	-2.65225800	-0.98412000
H	-11.92479300	-1.93791700	0.18539400
H	-11.66223800	-1.16646200	-1.35842900
H	-6.50165600	-3.20986000	-1.62712800
H	-5.85962400	-1.80162400	0.58686800
H	-7.74496300	-1.92579600	3.32706000
H	-9.04040700	-3.05813300	3.72821500
H	-7.53615200	-3.64450700	3.04545100
H	-9.08522600	-4.83523500	1.50466300
H	-10.51512400	-3.97412100	2.03201500
H	-10.08903500	-3.99858600	0.33667500
H	-7.50076100	4.07322200	-3.44179500
H	-10.65567200	2.47330300	-3.61733500
H	-6.64395600	3.13466000	1.64016800
H	-6.12363200	1.62116400	0.90978300
H	-12.24857300	2.76349800	0.29255400
H	-12.72405600	2.41931000	1.94213200
H	-11.06505900	2.88166500	1.58644200
H	-3.41724200	4.08610600	-0.08148600
H	-3.94678500	3.64181100	1.53801200
H	-2.36528800	1.95938000	0.85374700
H	-3.93766300	1.21822700	0.83772100
H	-3.98132700	1.84708600	-1.72267600
H	-2.27747400	2.13249800	-1.53790500
H	-2.52605000	-0.10316300	-2.34391000
H	-2.06844700	-0.27934700	-0.64966600
H	-5.81336500	-1.11895300	-1.77389700

Disclaimer/Publisher's Note: The statements, opinions and data contained in all publications are solely those of the individual author(s) and contributor(s) and not of MDPI and/or the editor(s). MDPI and/or the editor(s) disclaim responsibility for any injury to people or property resulting from any ideas, methods, instructions or products referred to in the content.
